# Rutin attenuates ensartinib-induced hepatotoxicity by non-transcriptional regulation of TXNIP

**DOI:** 10.1007/s10565-024-09883-4

**Published:** 2024-05-24

**Authors:** Wentong Wu, Jinjin Li, Yiming Yin, Yourong Zhou, Xiangliang Huang, Yashi Cao, Xueqin Chen, Yunfang Zhou, Jiangxia Du, Zhifei Xu, Bo Yang, Qiaojun He, Xiaochun Yang, Yuhuai Hu, Hao Yan, Peihua Luo

**Affiliations:** 1 Center for Drug Safety Evaluation and Research of Zhejiang University, College of Pharmaceutical Sciences, Zhejiang University, 866 Yuhangtang Road, Zijingang Campus, Zhejiang, 310058 Hangzhou China; 2 Department of Oncology, Affiliated Hangzhou Cancer Hospital, Zhejiang University School of Medicine, Key Laboratory of Clinical Cancer Pharmacology and Toxicology Research of Zhejiang Province, Hangzhou, 310002 China; 3 Cancer Center, Zhejiang University, Hangzhou, 310058 China; 4 The Laboratory of Clinical Pharmacy, the Sixth Affiliated Hospital of Wenzhou Medical University, The People’s Hospital of Lishui, Lishui, 323020 China; 5 Center for Medical Research and Innovation in Digestive System Tumors, Ministry of Education, the Second Affiliated Hospital, Zhejiang University School of Medicine, Hangzhou, 310017 China; 6 Institute of Pharmacology & Toxicology, College of Pharmaceutical Sciences, Zhejiang University, Hangzhou, 310058 China; 7 School of Medicine, Hangzhou City University, Hangzhou, 310015 China; 8 Innovation Institute for Artificial Intelligence in Medicine of Zhejiang University, Hangzhou, 310018 China; 9 Innovation Institute of Hangzhou Yuhong Pharmatech Co.,LTD, Hangzhou, 310018 China; 10 Department of Pharmacology and Toxicology, Hangzhou Institute of Innovative Medicine, College of Pharmaceutical Sciences, Zhejiang University, Hangzhou, 310018 China; 11 Key Laboratory of Clinical Cancer Pharmacology and Toxicology Research of Zhejiang Province, Affiliated Hangzhou Cancer Hospital, Zhejiang University School of Medicine, Hangzhou, 310002 China

**Keywords:** Ensartinib, Apoptosis, TXNIP, Hepatotoxicity, Rutin

## Abstract

Ensartinib, an approved ALK inhibitor, is used as a first-line therapy for advanced ALK-positive non-small cell lung cancer in China. However, the hepatotoxicity of ensartinib seriously limits its clinical application and the regulatory mechanism is still elusive. Here, through transcriptome analysis we found that transcriptional activation of TXNIP was the main cause of ensartinib-induced liver dysfunction. A high TXNIP level and abnormal TXNIP translocation severely impaired hepatic function via mitochondrial dysfunction and hepatocyte apoptosis, and TXNIP deficiency attenuated hepatocyte apoptosis under ensartinib treatment. The increase in TXNIP induced by ensartinib is related to AKT inhibition and is mediated by MondoA. Through screening potential TXNIP inhibitors, we found that the natural polyphenolic flavonoid rutin, unlike most reported TXNIP inhibitors can inhibit TXNIP by binding to TXNIP and partially promoting its proteasomal degradation. Further studies showed rutin can attenuate the hepatotoxicity of ensartinib without antagonizing its antitumor effects. Accordingly, we suggest that TXNIP is the key cause of ensartinib-induced hepatotoxicity and rutin is a potential clinically safe and feasible therapeutic strategy for TXNIP intervention.

**Graphical Abstract:**

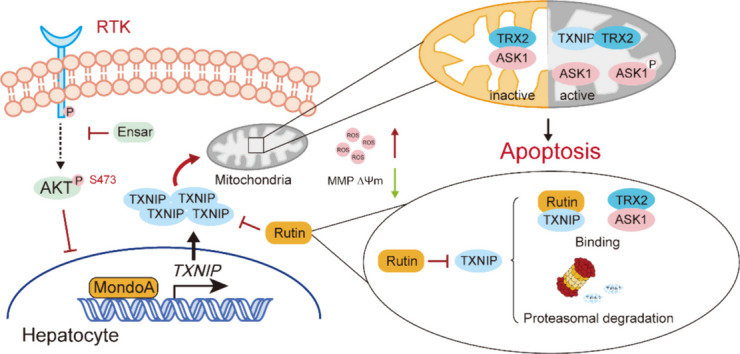

**Supplementary Information:**

The online version contains supplementary material available at 10.1007/s10565-024-09883-4.

## Introduction

Chromosomal rearrangements encoding the anaplastic lymphoma kinase (ALK) gene in non-small cell lung cancer (NSCLC) can be detected in 3-8% of cases (Schneider et al. 2023). ALK tyrosine kinase inhibitors (TKIs) are the first-line therapy for the treatment of advanced NSCLC with ALK rearrangements (Fukui et al. 2022). Crizotinib was the first ALK-TKI to significantly improve patient prognosis. However, challenges have arisen due to point mutations associated with crizotinib resistance. The potency of ensartinib, a novel aminopyridine-based small molecule, is ten times greater than that of crizotinib in inhibiting the growth of ALK-positive lung cancer cell lines and effectively suppressing ALK fusions associated with resistance (Horn et al. 2018; Zhang et al. 2022). However, adverse drug reactions associated with its use warrant consideration, with the most common reactions including rash, elevated alanine transaminase (ALT), and elevated aspartate transaminase (AST) (Zhou et al. 2023). Of particular concern is the potential of ensartinib to induce drug-related hepatotoxicity. Safety summaries from five clinical studies indicated a higher incidence of ALT and AST elevation in patients receiving ensartinib treatment, with some patients experiencing grade 3-4 adverse reactions (Yang et al. 2020; Ai et al. 2021; Horn et al. 2021; Ma et al. 2022; Luo et al. 2023). The mechanism of ensartinib-induced hepatotoxicity remains unclear, and effective intervention strategies are lacking, underscoring a significant unmet clinical need that requires resolution. Therefore, it is important to study the mechanism of ensartinib-induced hepatotoxicity for the safer clinical application of ensartinib.

Thioredoxin-interacting protein (TXNIP) is a crucial physiological inhibitor of the thioredoxin (TRX) redox system (Han et al. 2018). TXNIP possesses an α inhibitory domain that interacts with cytoplasmic TRX and mitochondrial TRX (in its active form), reducing their activity and thereby regulating cellular redox signal transduction. Under physiological conditions, TXNIP is mainly localized in the cell nucleus, and translocation from the nucleus to the cytoplasm is relatively difficult (Choi and Park 2023). However, in the presence of excessive reactive oxygen species (ROS), TXNIP is upregulated, translocates to mitochondria, and forms a complex with TRX2, promoting mitochondria-mediated cell apoptosis and causing various types of cellular damage. Additionally, TXNIP has been reported to induce cell cycle arrest in the G0/G1 phase, exerting anti-proliferative effects (Schulze et al. 2002), and TXNIP overexpression increases the susceptibility of myocardial cells, fibroblasts and pancreatic β-cells (Minn et al. 2005) to apoptosis (Chen et al. 2008).

In this study, we elucidated the mechanism of ensartinib-induced hepatotoxicity, wherein ensartinib upregulates TXNIP levels, subsequently triggering mitochondria-dependent apoptosis in hepatocytes. Conversely, rutin could negatively regulate TXNIP. We confirmed that rutin can directly interact with TXNIP. While rutin may modulate ROS-induced apoptosis by inhibiting the functional activity of TXNIP, rutin administration could also promote the ubiquitination of TXNIP and its subsequent proteasomal degradation. This finding indicated that rutin may impact TXNIP through multiple pathways in hepatocytes, suggesting a potential therapeutic strategy for mitigating ensartinib-induced hepatotoxicity.

## Materials and methods

### Animal experiments, biochemical assessment and histology

C57BL/6J male mice aged 6 weeks were supplied by Beijing Vital River Laboratory. The mice were housed in a facility under a 12-hour light/dark cycle and constant environmental conditions with temperatures between 21-23 °C and humidity levels of 40-70%. Following a 7-day acclimatization period under standard laboratory conditions, the mice were randomly allocated to various groups. In the study of ensartinib-induced liver toxicity, mice received 60 mg/kg or 120 mg/kg of ensartinib dissolved in 0.5% CMC-Na, and treated by gavage every day for 4 weeks (with 7 mice in each group). To evaluate the potential of rutin as a treatment for liver damage caused by ensartinib, male mice were administered 120 mg/kg ensartinib and/or 10 mg/kg rutin dissolved in 0.5% CMC-Na every day for 4 weeks (with 6 mice per group). As a control, a separate group of mice were given 0.5% CMC-Na by gavage. After 4 weeks of drug treatment, the mice were fasted overnight and sacrificed through cervical dislocation under anesthesia.

Under isoflurane anesthesia, blood drawn from the retro-orbital plexus of the mice that had settled for 2 hours was then centrifuged at 3500 × g for 15 minutes. Next, the supernatant was collected for analysis. The ALT, AST and alkaline phosphatase (ALP) levels of the supernatant were measured by a chemical analyzer (XN-1000V, Sysmex). Liver tissues were sent to HaoKe Biotechnology Co., Ltd. for hematoxylin and eosin (H&E) staining, Masson’s trichrome staining, and Sirius red staining. Panoramic view of tissue staining sections were observed and captured under a pathological section scanner (HS6, SUNNY INSTRUMENT CO., LTD) and the magnification of representative images were 100× magnification or 200× magnification.

### Primary hepatocyte isolation

Primary hepatocytes from 6- to 8-week-old male C57BL/6J mice were centrifuged by the collagenase perfusion and gradient method. Primary hepatocytes from mice were digested by infusion in IV collagenase solution (17104019, Sigma–Aldrich) dissolved in HBSS (14065056, Gibco). Next, the cells that had been isolated were sieved through a 70-mm cell strainer and subsequently cultured in dishes precoated with type I collagen (C8065, Solarbio).

### Cell culture

HL-7702 cells were obtained from Guangzhou Jennio Biotech Co., Ltd. Primary hepatocytes were extracted from 6- to 8-week-old male C57BL/6J mice. The AML12 cell line was generously supplied by the Stem Cell Bank at the Chinese Academy of Sciences. Additionally, HEK293T cells used for cellular thermal shift assay (CETSA) were supplied by the Institute of Biochemistry and Cell Biology. NCI-H3122 cells were originally obtained from Wuhan Procell Life Science&Technology Co., Ltd. cultured in specialized cell culture medium for NCI-H3122 (PM150110, Procell). Primary hepatocytes and AML12 cells were cultured in DMEM (10569010, Gibco), while HL-7702 cells were maintained in RPMI-1640 (21870076, Gibco) supplemented with 10% fetal bovine serum (16140071, Gibco) and penicillin/streptomycin in a humidified atmosphere with 5% CO_2_ at 37 °C. Routine testing was consistently performed on all cell lines to confirm the absence of mycoplasma contamination.

### Materials

Ensartinib (Ensar; T37585), brigatinib (Briga; T3621), crizotinib (Crizo; T1661), alectinib (Alec; T1936), ceritinib (Ceri; T1791), lorlatinib (Lorla; T3061), metformin (T8526), verapamil (Vera; T20656) and rutin (T0795) were purchased from Topscience. The natural products utilized in the drug screening were obtained from Nantong Jingwei Biotechnology Co., Ltd. Details regarding these products are presented in Table S1. Z-VAD-FMK (C1202) was obtained from Beyotime. Chloroquine (CQ; C6628) and MG132 (M8699) were obtained from Sigma–Aldrich. The antibodies used are shown in Table S2. Throughout the experiments (if not stated otherwise), HL-7702 cells were treated with 2 μM ensartinib for the indicated time periods or with 0, 1, 2 or 4 μM ensartinib for 36 h. In selected samples, 20 μM Z-VAD-FMK, 10 μM CQ, 10 μM MG132, 2.5 μM brigatinib, 2 μM crizotinib, 1.5 μM alectinib, 2 μM ceritinib, 1.5 μM lorlatinib, 0.5 mM metformin, 50 μM verapamil and 5 μM rutin were used.

### RNA-seq transcriptome analysis

Using TRIzol reagent, RNA was extracted from HL-7702 cells treated with either DMSO or 2 μM ensartinib. RNA-seq analysis was conducted by LC-Bio, and the resulting data were stored in the NCBI Gene Expression Omnibus database (GSE249370).

### Western blot analysis

Protein was isolated by employing a lysis buffer mixture consisting of of 150 mM NaCl, 50 mM Tris-HCl, 2 mM EGTA, 2 mM EDTA, 25 mM sodium glycerophosphate, 25 mM NaF, 0.3% Triton X-100, 0.3% NP-40, 0.3% leupeptin, 0.1% NaVO_3_, and 0.1% PMSF. Lysates were subjected to SDS–PAGE at concentrations of 8%, 10%, or 12%, transferred to PVDF membranes provided by Millipore Corporation and incubated with a 3% solution of bovine serum albumin. Subsequently, the PVDF membranes were washed three times in PBS containing 0.1% Tween-20 (T-PBS) and incubated with primary antibodies followed by three washes in T-PBS and incubation for 1 h with secondary antibodies. Following three washes in T-PBS, band detection was performed using the ECL-Plus Kit (P2300, NCM Biotech), and an Amersham Imager 600 (General Electric Company) was used for visualization. The primary antibodies utilized for western blot analysis are listed in Supplementary Table S2. HRP-labeled secondary antibodies (GAR007, GAM007, 1:1000) were obtained from LiankeBio. The primary antibodies used include: beta Actin (Diagbio, db7283, 1:2000), TXNIP (Huabio, ET1705-72, 1:1500), Cleaved PARP (Huabio, ET1608-10, 1:1500), Cleaved PARP (Abcam, ab32064, 1:1500), cleaved caspase-3 (Asp175) (Cell Signaling Technology, #9661, 1:2000), phospho-ASK1 (ABclonal, AP1215, 1:1000), γ-H2AX (Cell Signaling Technology, #9718, 1:2000), Ub (FL-76) (Santa Cruz Biotechnology, sc-9133, 1:1000), p-Akt (S473) (ABclonal, AP1208, 1:1000), LC3 A/B (Medical & Biological Laboratories, M186-3, 1:2500). We used Image J to perform a statistical analysis of the optical density of the western blot experiment. Specifically, the ratio was obtained by dividing the protein optical density by the ACTB optical density, and then quotient the ratio with the control ratio.

### SRB (Sulforhodamine B) staining

To assess the cell survival rate, sulforhodamine B (SRB; S1402, Sigma–Aldrich) was utilized. In brief, in 96-well plates, the cells were fixed with 10% trichloroacetic acid (T104257, Aladdin) and treated with 4 mg/mL SRB for 30 minutes. Subsequently, the plates were washed with 1% acetic acid (1000218, Sinopharm). The residual SRB dye was dissolved in 10 mM unbuffered Tris base (1115KG001, Biofroxx). The absorbance at 515 nm was measured using a multiscan spectrophotometer (Tecan).

### Quantitative polymerase chain reaction (qPCR)

Following treatment, the cells were collected, and RNA was isolated using the FastPure Cell/Tissue Total RNA Isolation Kit V2 (RC112, Vazyme) according to the manufacturer’s instructions. The RNA was then uniformly reverse transcribed into cDNA for use in subsequent qPCR experiments. The qPCR amplification was performed in two steps, beginning with an initial 3-minute phase at 95 °C, followed by 39 cycles of 95 °C for 3 seconds and 60 °C for 31 seconds each. Gene expression fold changes were determined using the comparative threshold cycle (Ct) method and the 2^−(ΔΔCt)^ formula. The detailed primer sequences, provided by Youkang Biotechnology Co., Ltd., were shown as below:

TXNIP forward, 5'- GAGTACCTGCGCTATGAAGAC -3',

TXNIP reverse, 5'- TTTGAAGGATGTTCCCAGAGG -3',

IDH1 forward, 5'- CAAGTGACGGAACCCAAAAG -3',

IDH1 reverse, 5'- ACCCTTAGACAGAGCCATTTG -3',

DUSP6 forward, 5'- CGACTGGAACGAGAATACGG -3',

DUSP6 reverse, 5'- CAGCCAAGCAATGTACCAAG -3',

HNF4A forward, 5'- TGGACAAAGACAAGAGGAACC -3',

HNF4A reverse, 5'- ATAGCTTGACCTTCGAGTGC -3',

ACTB forward, 5'- CACCATTGGCAATGAGCGGTTC -3',

ACTB reverse, 5'- AGGTCTTTGCGGATGTCCACGT -3'.

### Transfection of siRNA oligonucleotides

Transfection of siRNA oligonucleotides was achieved using Oligofectamine™ transfection reagent (12252011, Invitrogen). The medium in a 6-well plate was removed and replaced with the prepared transfection solution containing Opti-MEM™ (31985070, Gibco). After 6 hours of transfection, the transfection medium was replaced with complete culture medium, and drug treatments were administered as per the experimental design. The following oligonucleotides were provided by GenePharma:

siRNA#1 targeting *TXNIP*, 5'- GGCAAUCUCCUGGGCCUUAdTdT-3',

siRNA#2 targeting *TXNIP*, 5'- AGAAUACAUGUUCCCGAAGdTdT-3',

siRNA targeting *DUSP6*, 5'- AAUGUCAUAGGCAUCGUUCdTdT-3',

siRNA targeting *HNF4A*, 5'- CCACAUGUACUCCUGCAGAdTdT-3',

siRNA targeting *IDH1*, 5'- CCUUUGUAUCUGAGCACCAdTdT-3',

siRNA negative control, 5'- UUCUCCGAACGUGUCACGUdTdT-3'.

### Flow cytometry analysis of Annexin V-PI staining

The proportion of apoptotic cells was detected with an Apoptosis Assay Kit I (C1062L, Beyotime). Briefly, cells were processed for Annexin V-PI staining at the indicated times. A total of 1 × 10^4^ cells per sample were measured by a flow cytometer (BD Biosciences). The gating strategies for classification were documented using BD CellQuest Pro software.

### JC-1 staining

To assess mitochondrial membrane potential (MMP), a JC-1 staining kit (C2005, Beyotime) was used. After treatment, cells were collected and rinsed with PBS. The cells were then stained with 5 μM JC-1, as provided in the kit, for 30 minutes at 37 °C in the dark. As a positive control, 10 μM CCCP was applied 20 minutes prior to the assay. For flow cytometry, 1 × 10^4^ cells were prepared and measured using a flow cytometer (BD Biosciences). For fluorescence microscopy, nuclei were stained with DAPI (D212, Dojindo), and images for every sample (400× magnification) were captured with a fluorescence microscope (IX81-FV1000, Olympus).

### Reactive oxygen species detection

Reactive oxygen species (ROS) were measured using a Reactive Oxygen Species Assay Kit (S0033S, Beyotime). Before loading with the DCFH-DA probe, 50 μg/mL Rosup was added to the positive control wells for pretreatment for 30 min. The cells were incubated in serum-free medium containing the DCFH-DA probe and diluted at a 1:1000 ratio for 20 minutes according to the instructions. For flow cytometry analysis of ROS, 1 × 10^4^ cells were analyzed by a BD FACSCalibur™ flow cytometer (BD Biosciences). Fluorescence images for every sample (200× magnification or 400× magnification) were captured after staining the nuclei with DAPI (D212, Dojindo) with a fluorescence microscope (IX81-FV1000, Olympus).

### MitoTracker staining

To assess the amount of mitochondria, HL-7702 cells were subjected to selective staining using the deep red fluorescent marker MitoTracker (M22426, Invitrogen). These cells were grown on the top of a Petri dish that was filled with a suitable growth solution. Upon reaching the required confluency, the existing medium was replaced with a staining solution preheated to 37 °C containing a 100 nM concentration of the MitoTracker probe following the provided instructions. Under conditions conducive to hepatocyte growth, the cells were incubated for 25 minutes, and then images for every sample (400× magnification) were captured using a fluorescence microscope (IX81-FV1000, Olympus).

### MitoSOX staining

Mitochondrial ROS were measured with a MitoSOX staining kit (M36008, Invitrogen). Before the experiment, cells were plated on a 25-mm cover slip for 24 hours and treated with ensartinib. According to the instructions, the cells were incubated with MitoSOX at a concentration of 50 μM for 45 minutes. Images for every sample (400× magnification) were captured under a fluorescence microscope (IX81-FV1000, Olympus).

### TUNEL staining

Liver specimens were initially fixed thoroughly using formalin (F8775, Sigma–Aldrich), followed by paraffin embedding and sectioning. These sections were then deparaffinized and subjected to proteinase K treatment (ST532, Beyotime) for 15 minutes. The slides were subsequently rinsed three times in PBS, followed by the addition of 50 μL of TUNEL reaction mix (C1088, Beyotime) and incubation for 60 minutes. Then, the nuclei were stained with DAPI (D212, Dojindo). Finally, the stained sections were imaged for every sample (200× magnification) using a fluorescence microscope (IX81-FV1000, Olympus).

### Immunofluorescence

Both cells and liver tissue sections were fixed with 4% paraformaldehyde for 15 minutes (P6148, Sigma–Aldrich), followed by permeabilization with 0.3% Triton X-100 (1139ML100, Biofroxx) for 5 minutes. After three PBS washes, the cells were blocked in 4% bovine serum albumin (BSA; B2064, Sigma–Aldrich), and the liver tissues were blocked with 5% goat serum (S9070, Solarbio) for 30 minutes. The samples were then incubated overnight with the target antibody at 4 °C. After another three PBS washes, the cells were treated with Alexa Fluor 488- or Alexa Fluor 568-conjugated secondary antibodies (A21202, A10042, A31573, Thermo Fisher Scientific) for 1 hour, followed by DAPI staining (D212, Dojindo). The primary antibodies used included anti-TXNIP (Huabio, ET1705-72, 1:200) and anti-Tomm20 (Santa Cruz Biotechnology, sc-17764, 1:200). Fluorescence images for every sample (200× magnification or 400× magnification) were captured using a fluorescence microscope (IX81-FV1000, Olympus). The antibodies used for immunofluorescence are listed in Table S2.

### Immunohistochemical staining

The immunohistochemistry process began with deparaffinization of the tissue sections in xylene, followed by rehydration using graded alcohol. Next, the sections were treated with 3% H_2_O_2_ (PV-6001, ZSGB-BIO) for 5 minutes and subsequently blocked for 30 minutes using 5% goat serum (16210064, Gibco). After this blocking step, the liver tissues were incubated overnight with specific antibodies against TXNIP (Huabio, ET1705-72, 1:200) and c-caspase-3 (Asp175) (Cell Signaling Technology, 9661, 1:200). Following three PBS washes, the sections were further incubated with an enzyme-conjugated secondary antibody (PV-6001, ZSGB-BIO) for 60 minutes. Staining was completed using DAB chromogen (ZLI-9017, ZSGB-BIO), and the nuclei were counterstained with hematoxylin (C0107, Beyotime) for a brief period of 3 seconds. The resulting images for every sample (200× magnification) were scanned with a pathological section scanner (HS6, SUNNY INSTRUMENT CO., LTD). The specific antibodies utilized for staining are detailed in Table S2.

### ATP measurement

ATP levels were measured using an ATP detection kit (S0027, Beyotime). Following treatment with ensartinib, the cells were lysed using the ATP lysis buffer provided in the kit. After centrifugation, the supernatant was collected from the cell lysates. The ATP assay solution was then prepared according to the kit instructions and added to the samples for 3-5 minutes at room temperature. Samples or standards, 20 μL each, were dispensed into a black bottom 96-well plate (165305, Thermo Fisher Scientific). Relative light units (RLU) were quantified using a multifunctional microplate detector (Tecan).

### Cellular thermal shift assay (CETSA)

CETSA procedures were performed based on previous studies (Jafari et al. 2014). Briefly, 100 μL of 293T cell lysate was mixed with rutin, silibinin, or salidroside at a final concentration of 5 μM. In the control group, the cell lysate was coincubated with DMSO, the solvent used for rutin. The mixture was incubated at various temperatures (42.0 °C, 42.5 °C, 43.7 °C, 45.6 °C, 48.2 °C, 53.2 °C, 55.8 °C, 58.4 °C, 60.3 °C, 61.5 °C, and 62.0 °C) for 6 minutes. To obtain the supernatant, all the mixtures were centrifuged at 12,000 rpm for 15 minutes. SDS–PAGE was used to analyze the expression of TXNIP in the supernatant.

### Assessment of DNA damage using the comet assay

For the single-cell gel electrophoresis (SCGE) assay, as described by Velma and Tchounwou (Velma and Tchounwou 2013), HL-7702 cells were suspended in ice-cold PBS at a concentration of 1 × 10^5^ cells/mL, for a total volume of 1 mL. Images for every sample (400× magnification) were captured using a fluorescence microscope (IX81-FV1000, Olympus). For the analysis of comet experimental results, we use Image J to analyze the tail length.

### Molecular docking

The crystallographic structure of TXNIP (PDB ID 4II1) was sourced from the Protein Data Bank, while the molecular structure of rutin (PubChem CID 5280805) was retrieved from the PubChem database. Preparation of the TXNIP and rutin structures for docking was performed using AutoDockTools 1.5.6. The docking parameters were established as outlined in a previous study (Ferreira et al. 2015). The optimal docking configurations were visualized using PyMOL software.

### Statistical analysis

The data are presented as the means ± standard deviations (SDs). Differences were deemed significant when the *P* value was less than 0.05. To assess significant differences between two groups, Student’s *t* test was employed, while one-way ANOVA was used for comparisons involving three or more groups. The data were analyzed using Microsoft Excel and GraphPad Prism software. Comprehensive statistical details, including the precise number of mice used, are provided in the legends of the figures for each experiment. No data were excluded from any of the experiments.

## Results

### Ensartinib induced hepatotoxicity *in vivo*

**Fig. 1:**
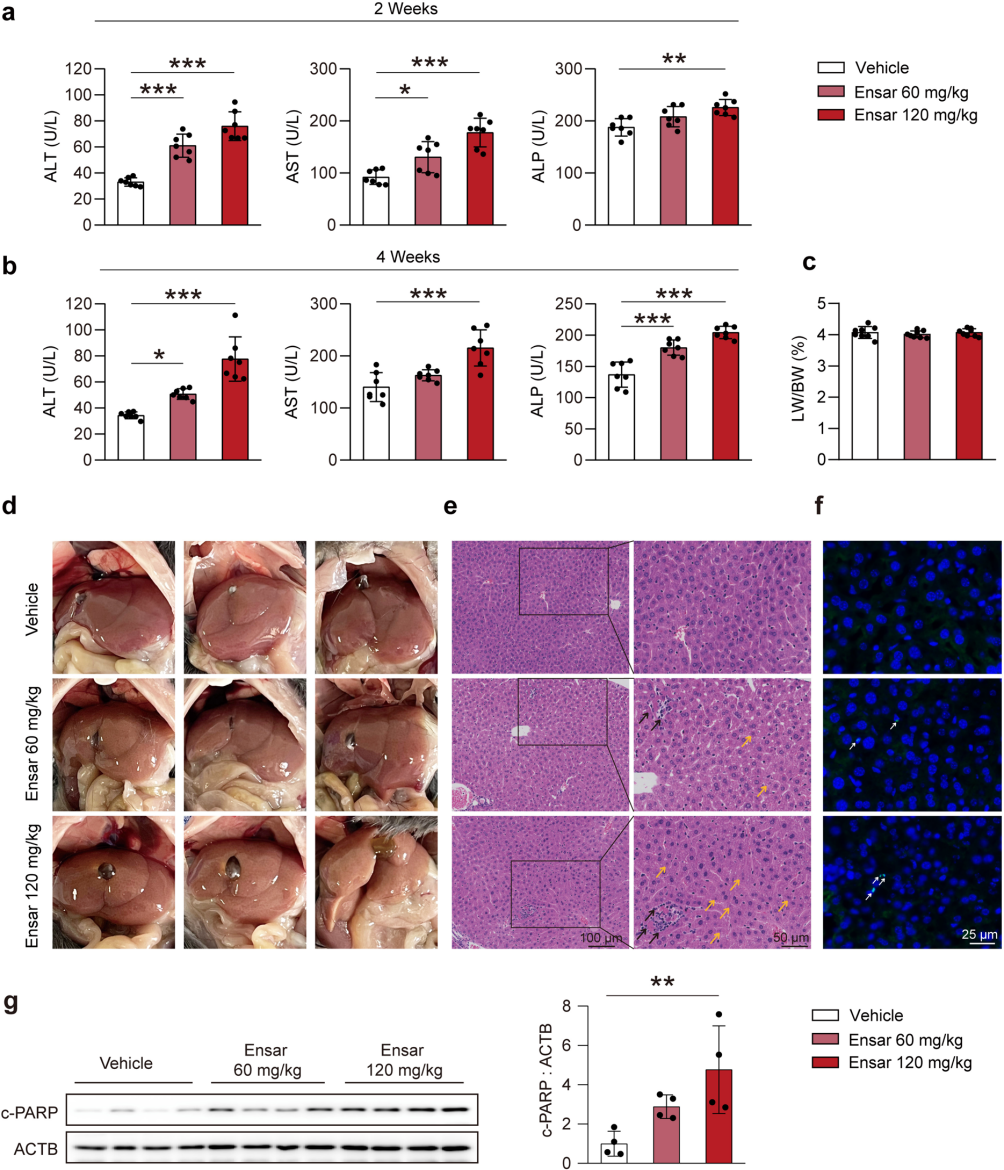
Ensartinib induced hepatotoxicity. **(a-g)** Male C57BL/6J mice were subjected to treatment with 0.5% CMC-Na, or ensartinib at doses of 60 mg/kg/day or 120 mg/kg/day for a duration of 4 weeks, with each treatment group comprising 7 mice. **(a-b)** Serum levels of ALT, AST, and ALP were measured and analyzed at both 2-week and 4-week intervals. **(c)** The levels of LW/BW of mice were analyzed. **(d)** Representative images of liver. **(e)** Representative H&E staining images. The scale bar measures 100 μm at 100× magnification and 50 μm at 200× magnification. The yellow arrows: shrunken nucleus; the black arrows: infiltration of inflammatory cells. **(f)** Representative TUNEL staining images of liver tissues (original magnification: 200×) with a scale bar indicating 25 μm. **(g)** The expression level of c-PARP in liver tissues (*n* = 4). Data were expressed as mean ± SD. *P < 0.05, **P < 0.01, ***P < 0.001. One way ANOVA followed by Dunnett T3 post hoc test for **(a)**, **(b)**, **(c)** and **(g)**. Ensar, ensartinib

Hepatotoxicity is a common and severe issue in the clinical application of tyrosine kinase inhibitors (TKIs) for anticancer treatment. To better understand the specific mechanism by which ensartinib causes hepatotoxicity in clinical settings, for a period of 4 weeks, C57BL/6J mice were treated daily with either 0.5% CMC-Na or ensartinib (administered at doses of 60 mg/kg or 120 mg/kg) via gavage. Serum samples were collected at the 2-week and 4-week marks during the administration period. Our findings revealed a significant increase in alanine transaminase (ALT), aspartate transaminase (AST), and alkaline phosphatase (ALP) following ensartinib administration (Fig. 1a, b). Despite the significant changes observed in liver enzymes, the ratio of liver weight to body weight remained relatively stable, with no noticeable alterations (Fig. 1c). Compared to those of the control group, the livers of the mice treated with ensartinib exhibited a lighter color, indicating apparent abnormalities (Fig. 1d). This finding was further confirmed by hematoxylin and eosin (H&E) staining, which revealed nuclear shrinkage (yellow arrows) and infiltration of inflammatory cells (black arrows) in the ensartinib-treated group (Fig. 1e). Furthermore, both Masson’s trichrome staining and Sirius red staining provided evidence of significant liver fibrosis induced by ensartinib treatment (Fig. S1a, b).

Apoptosis is predominantly considered to be the main cause of hepatocyte damage in drug-induced liver injury (Andrade et al. 2019; Iorga et al. 2017). To determine the cause of hepatotoxicity and hepatocyte death, we conducted terminal deoxynucleotidyl transferase dUTP nick end labeling (TUNEL), a classic apoptosis assay used to measure the population of apoptotic cells in liver tissue. After ensartinib stimulation, significant colocalization of green fluorescence with the nuclear area of hepatocytes was observed, indicating DNA fragmentation—a signal of late apoptosis (Fig. 1f). Furthermore, western blot results revealed a dose-dependent increase in the protein levels of apoptosis markers, specifically, cleaved-poly (ADP-ribose) polymerase (c-PARP), following ensartinib treatment *in vivo* (Fig. 1g). These results suggested that treatment with ensartinib caused severe hepatic impairment by increasing hepatocyte apoptotic death.

### Ensartinib induced apoptosis in hepatocytes

Next, we validated the hepatotoxic effects of ensartinib. The human hepatocyte cell line HL-7702 was stimulated with gradient concentrations of ensartinib (0, 0.25, 0.5, 1, 2, or 4 μM) for 24, 48, or 72 hours. We determined the survival rates of the cells using a sulforhodamine B (SRB) assay. Our findings revealed that ensartinib directly reduced the survival rate of hepatocytes, an effect that intensified with increasing treatment durations and drug concentrations (Fig. S2a). A decrease in the number of hepatocytes was also observed under a light microscope following stimulation with different concentrations of ensartinib. Compared with that in the control group, the morphology of the cells treated with ensartinib showed noticeable shrinkage (Fig. S2b). Collectively, these data confirm that ensartinib induces hepatic toxicity both *in vivo* and *in vitro*, which is consistent with our previous observations and clinical findings.

**Fig. 2:**
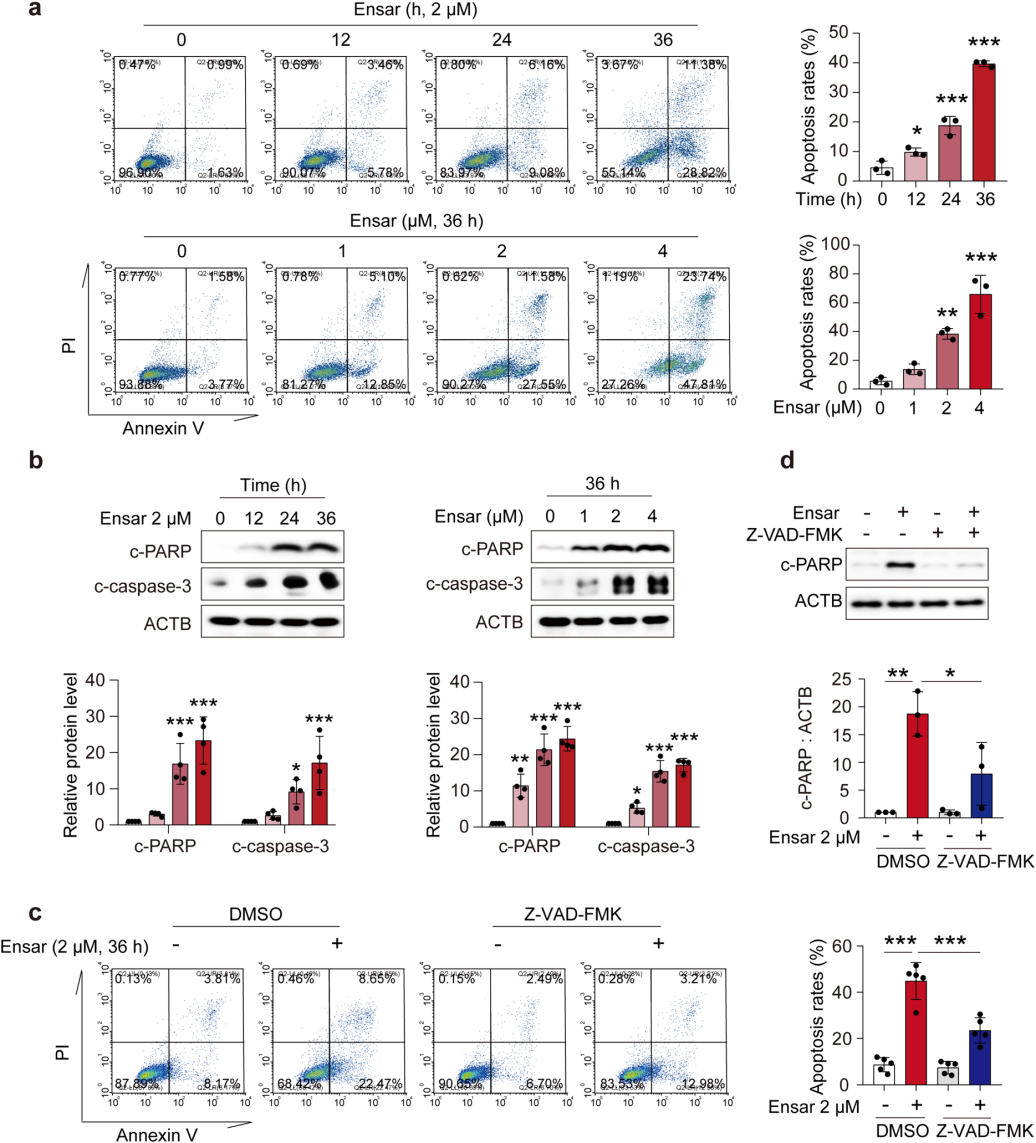
Ensartinib caused hepatocyte apoptosis. **(a-b)** HL-7702 cells underwent treatment with 2 μM ensartinib for durations of 12, 24, or 36 hours, and were also exposed to varying concentrations of 0, 1, 2, or 4 μM ensartinib for a period of 36 hours (*n* = 3). **(a)** The apoptosis rates of HL-7702 cells (*n* = 3). **(b)** The expression levels of c-PARP and c-caspase-3 in HL-7702 cells (*n* = 4). **(c-d)** HL-7702 cells underwent treatment with 2 μM of ensartinib and/or 20 μM of Z-VAD-FMK for a duration of 36 hours. **(c)** The apoptosis rates of HL-7702 cells (*n* = 5). **(d)** The expression level of c-PARP in HL-7702 cells (*n* = 3). Data were expressed as mean ± SD. *P < 0.05, **P < 0.01, ***P < 0.001. One way ANOVA followed by Dunnett T3 post hoc test for **(a)** and **(b)** or one way ANOVA followed by Tukey post hoc test for **(c)** and **(d)**. Ensar, ensartinib

To ascertain whether hepatocyte apoptosis contributes to the hepatotoxicity induced by ensartinib, we employed PI/Annexin V staining with flow cytometry to characterize apoptotic hepatocytes that had been stimulated with ensartinib (0, 1, 2, or 4 μM) or 2 μM ensartinib for 12, 24, or 36 hours. The results, shown in the upper right and lower right quadrants of the corresponding figure, showed a significant time-dependent and concentration-dependent increase in apoptosis rates (Fig. 2a). Western blot analysis further revealed that the protein levels of c-PARP and cleaved caspase 3 (c-caspase-3, Fig. 2b) increased with increasing concentrations of ensartinib and stimulation time. We also found that the common pancaspase inhibitor Z-VAD-FMK, when administered in combination with ensartinib, could restore the number and morphology of cells impaired by ensartinib treatment and relieve ensartinib-induced hepatocyte apoptosis, as evidenced by western blot and flow cytometry analysis (Fig. 2c, d, Fig. S2c). These results conclusively indicated that ensartinib causes caspase-dependent apoptosis in hepatocytes both *in vivo* and *in vitro*. Based on these findings, we conclude that the hepatotoxicity caused by ensartinib is due to the induction of hepatocyte apoptosis.

### Ensartinib induced DNA damage and mitochondrial dysfunction

**Fig. 3:**
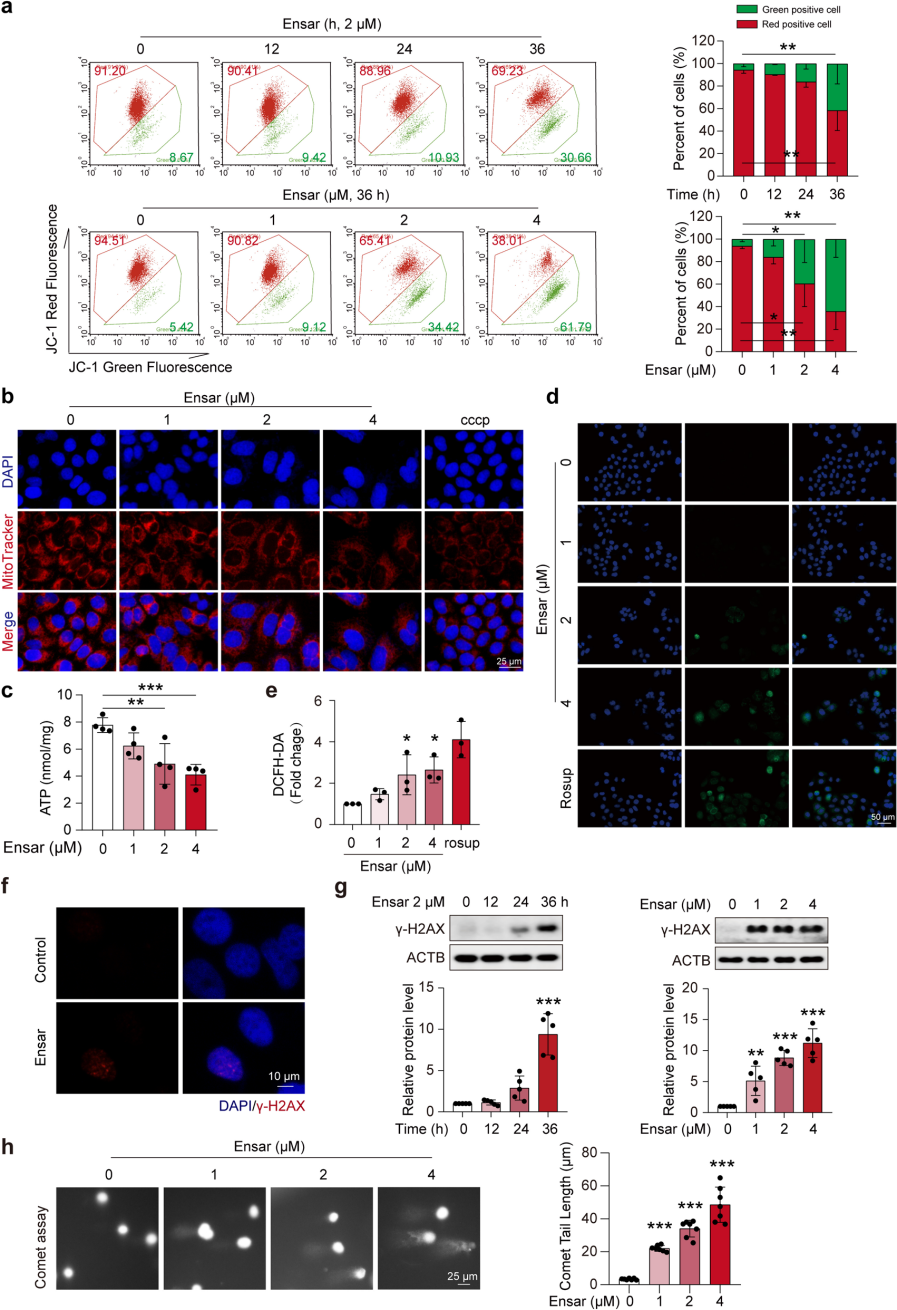
Ensartinib caused mitochondrial damage and DNA damage in hepatocytes. **(a)** HL-7702 cells underwent treatment with 2 μM of ensartinib for 12, 24, and 36 hours, as well as with varying concentrations of 0, 1, 2, and 4 μM ensartinib for 36 hours. Subsequently, mitochondrial membrane potential (MMP) was assessed (*n* = 3). **(b)** Representative images of MitoTracker red staining (original magnification: 400×) in HL-7702 cells treated with ensartinib at concentrations of 0, 1, 2, or 4 μM for a duration of 36 hours. Scale bar: 25 μm. **(c-e)** HL-7702 cells received treatment using ensartinib at concentrations of 0, 1, 2, or 4 μM for a period of 36 hours. **(c)** The ATP levels in HL-7702 cells (*n* = 4). **(d-e)** The ROS levels in HL-7702 cells. Representative images (original magnification: 200×) are shown. Scale bar: 50 μm (*n* = 3). Rosup was used as a positive control. **(f)** Representative images of immunofluorescence for γ-H2AX staining (original magnification: 400×) in HL-7702 cells following a 36-hour treatment with 2 μM ensartinib. Scale bar: 10 μm. **(g)** The expression levels of γ-H2AX in HL-7702 cells underwent treatment with 2 μM ensartinib for 12, 24, or 36 hours, and with doses of 0, 1, 2, or 4 μM ensartinib for 36 hours (*n* = 5). **(h)** HL-7702 cells were treated with 0, 1, 2 or 4 μM ensartinib for 36 h. The comet assay (original magnification: 400×) revealed the extent of DNA damage in hepatocytes (*n* = 6). Scale bar: 25 μm. Data were expressed as mean ± SD. *P < 0.05, **P < 0.01, ***P < 0.001. One way ANOVA followed by Dunnett T3 post hoc test for **(a)**, **(c)**, **(e)**, **(g)** and **(h)**. Ensar, ensartinib

Cell mitochondrial membrane potential (MMP), an indicator of mitochondrial apoptotic signaling pathways, decreases at the early stage of apoptosis (Bock and Tait 2020). To investigate changes in the MMP following ensartinib treatment, we performed JC-1 staining on hepatocytes. MMP alterations were reflected by the relative proportions of the measured red and green fluorescence. Our findings indicated that the MMP in hepatocytes decreased following ensartinib administration (Fig. 3a, Fig. S3a), suggesting increased mitochondrial membrane permeability. This result was further substantiated by the results of MitoTracker staining and ATP measurements, which were conducted to assess the impact of ensartinib on mitochondria. We observed dose-dependent decreases in mitochondrial mass (Fig. 3b) and ATP production (Fig. 3c) following ensartinib treatment. Mitochondrial damage can result in excessive intracellular accumulation of reactive oxygen species (ROS) (Zorov et al. 2014). Therefore, we sought to determine the overall and mitochondrial ROS levels using DCFH-DA and MitoSOX, respectively. The results from both the immunofluorescence assay and flow cytometry showed concentration-dependent increases in both overall and mitochondrial ROS levels after ensartinib stimulation (Fig. 3d, e, Fig. S3b).

Following ensartinib treatment, we observed an increase in the expression of γ-H2AX, a marker of DNA damage, which was increased in a concentration-dependent manner through immunofluorescence and western blot assays (Fig. 3f, g). This finding is typically indicative of apoptotic DNA damage caused by exogenous cell damage. To further investigate DNA damage, we conducted an alkaline single-cell gel electrophoresis (SCGE) assay, also known as the comet assay. The results showed a concentration-dependent increase in DNA damage, indicated by increased comet tail length, after ensartinib treatment (Fig. 3h). In conclusion, these findings suggest that ensartinib treatment can potentially induce apoptosis in hepatocytes through a mitochondria-mediated pathway.

### RNA-Seq analysis revealed that ensartinib-induced hepatocyte apoptosis is related to mitochondrial dysfunction and DNA damage

**Fig. 4:**
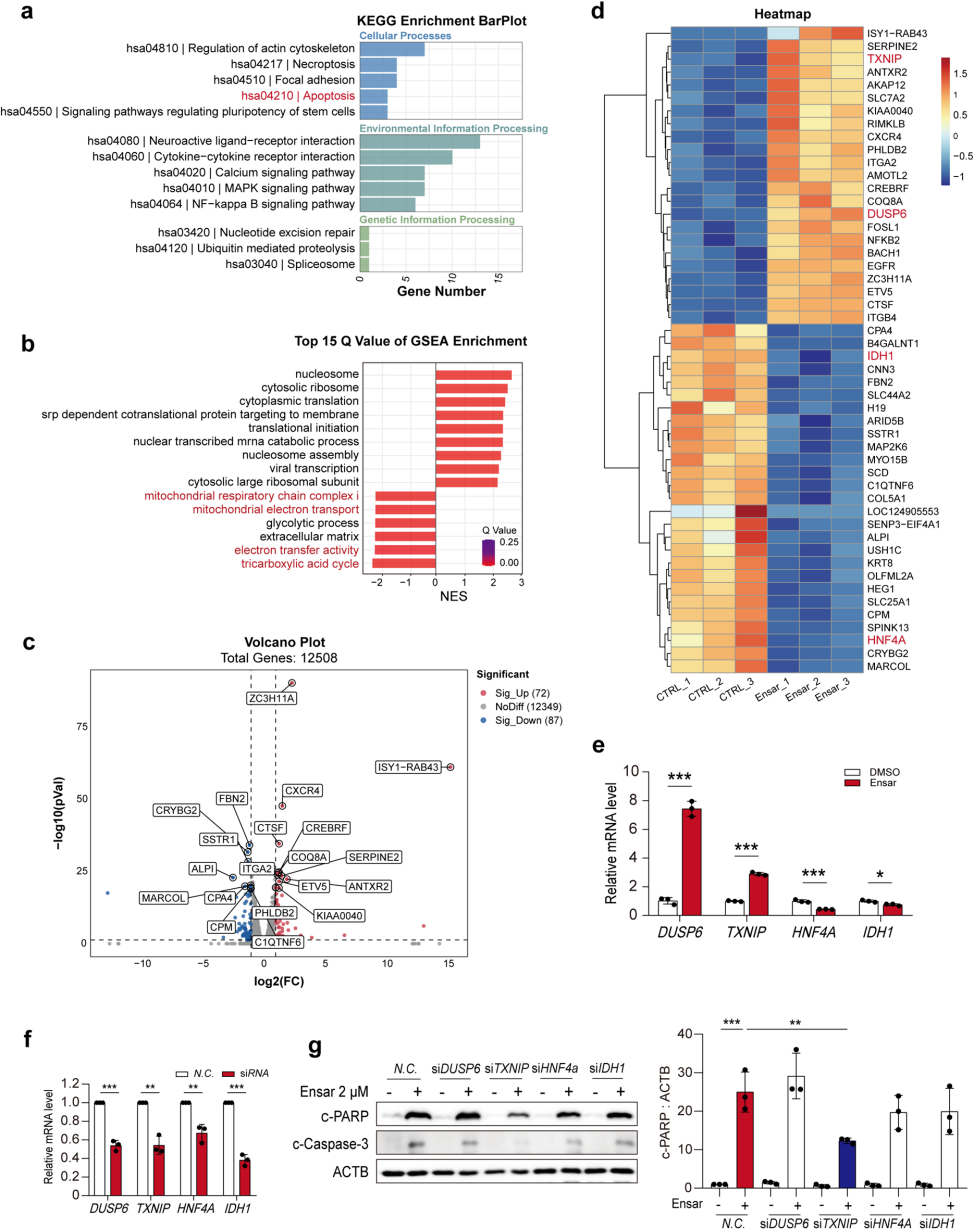
Transcriptomics showed the effect of ensartinib on hepatocyte signaling pathway. **(a-d)** HL-7702 samples from control and ensartinib groups were subjected to RNA-seq analysis (*n* = 3). **(a-b)** KEGG enrichment analysis and GSEA enrichment showing the most influential signaling pathway. **(c-d)** Volcano Plot and Heatmap showcasing the differentially expressed genes. **(e)** The mRNA expression levels of DUSP6, TXNIP, HNF4A, IDH1 in HL-7702 cells treated with 2 μM ensartinib for 36 h (*n* = 3). **(f-g)** NC transfected or DUSP6, TXNIP, HNF4A, IDH1-knockdown HL-7702 cells were treated with ensartinib for 36 h (*n* = 3). **(f)** Knockdown efficiency of siRNA (*n* = 3). **(g)** The expression levels of c-PARP and c-caspase-3 in HL-7702 cells (*n* = 3). Data were expressed as mean ± SD. *P < 0.05, **P < 0.01, ***P < 0.001. Unpaired two-sided Student’s *t* test for **(e)** and **(f)** or one way ANOVA followed by Tukey post hoc test for **(g)**. Ensar, ensartinib

Given the above results, we sought to identify potential factors contributing to the hepatotoxicity of ensartinib via RNA-Seq analysis. KEGG analysis revealed strong enrichment of apoptosis-related genes among these differentially expressed genes (Fig. 4a). Furthermore, GSEA enrichment analysis indicated downregulation of genes associated with the mitochondrial respiratory chain complex and mitochondrial electron transport, which is consistent with our previous findings (Fig. 4b). Transcriptome analysis revealed 72 upregulated and 87 downregulated genes (Fig. 4c). From these genes, we carefully selected genes associated with apoptosis or mitochondrial damage that exhibited differential expression post-ensartinib treatment, which led us to identify DUSP6 (Wang et al. 2021), TXNIP (Ao et al. 2021), HNF4A (Xu et al. 2022), and IDH1 (Shait Mohammed et al. 2022) as potential targets implicated in ensartinib-related hepatotoxicity (Fig. 4d). The *in vitro* mRNA expression levels mirrored the RNA-Seq analysis results, validating the reliability of the RNA-Seq findings (Fig. 4e). Subsequently, we employed a small interfering RNA (siRNA) to assess the impact of the expression of these genes on apoptosis levels in response to ensartinib. Due to the lack of related antibodies, we measured the silencing efficacy of each siRNA using qPCR and found that all of them were effective (Fig. 4f). For the two downregulated genes IDH1 and HNF4A, siRNA silencing did not induce HL-7702 cell apoptosis, and the combination of ensartinib did not further aggravate apoptosis, demonstrating that they were not the cause of ensartinib-induced hepatotoxicity. On the other hand, a reduced level of TXNIP was found to alleviate ensartinib-induced hepatocyte apoptosis, as evidenced by decreased protein levels of c-PARP and c-caspase-3 (Fig. 4g). In conclusion, our data suggest that ensartinib may induce hepatocyte apoptosis by triggering mitochondrial dysfunction, which is consistent with our previous findings. Moreover, TXNIP may be a key factor driving ensartinib-induced hepatotoxicity.

### TXNIP is responsible for ensartinib-induced hepatotoxicity

**Fig. 5:**
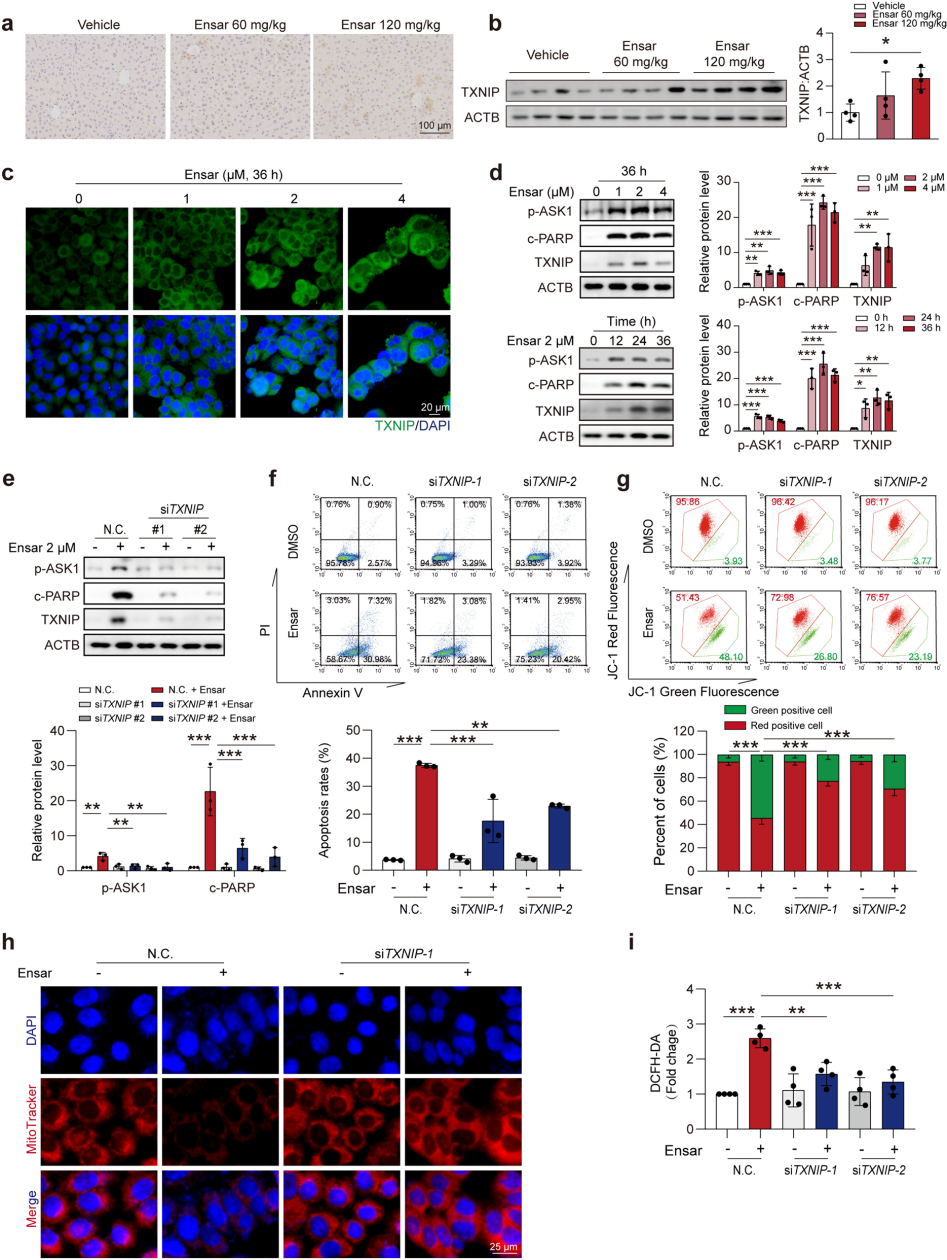
Ensartinib induces hepatocyte apoptosis by up-regulating TXNIP protein level. **(a)** C57BL/6J mice were administered a diet containing 0.5% CMC-Na, with ensartinib doses of either 60 mg/kg/day or 120 mg/kg/day for a duration of four weeks. Representive images (original magnification: 200×) of immunohistochemical staining for TXNIP in the liver tissues. Scale bar: 100 μm. **(b)** The expression levels of TXNIP in liver tissues (*n* = 4). **(c)** The localization and expression levels of TXNIP (original magnification: 200×) in HL-7702 cells treated with 2 μM ensartinib for 36 hours. Scale bar: 20 μm. **(d)** The expression levels of c-PARP and TXNIP in HL-7702 cells were treated with 0, 1, 2 or 4 μM ensartinib for 36 h or 2 μM ensartinib for 12, 24 or 48 h (*n* = 3). **(e-i)** NC transfected or TXNIP-knockdown HL-7702 cells were treated with or without 2 μM ensartinib for 36 h. **(e)** The expression level of c-PARP, TXNIP and p-ASK1 in HL-7702 cells (*n* = 3). **(f)** The apoptosis rates of HL-7702 cells (*n* = 3). **(g)** MMP of HL-7702 cells (*n* = 3). **(h)** Mitochondrial mass (original magnification: 400×) in HL-7702 cells. Scale bar: 25 μm. **(i)** ROS level measured by DCFH-DA staining in HL-7702 cells (*n* = 4). Data were expressed as mean ± SD. *P < 0.05, **P < 0.01, ***P < 0.001. One way ANOVA followed by Dunnett T3 post hoc test for **(b)** and **(d)** or one way ANOVA followed by Tukey post hoc test for **(e)**, **(f)**, **(g)** and **(i)**. Ensar, ensartinib

We hypothesized that the key event in ensartinib-driven hepatotoxicity might be increased TXNIP expression. Consistent with the results of the RNA-Seq analysis, TXNIP levels were significantly upregulated in a dose-dependent manner after ensartinib treatment both *in vivo* and *in vitro* (Fig. 5a-c). The protein level of TXNIP was consistent with that of c-PARP across different hepatocyte lines (Fig. 5d, Fig. S4a, b). Silencing TXNIP using siRNA decreased the levels of c-PARP and c-caspase-3, indicating a decreased apoptotic state (Fig. 5e). This finding was further corroborated by PI/Annexin V staining (Fig. 5f). Notably, TXNIP silencing ameliorated the mitochondrial damage and ROS levels induced by ensartinib treatment (Fig. 5g-i). Together, these results further confirm that TXNIP might be the main cause of the hepatotoxicity induced by ensartinib treatment and that a lower level of TXNIP could reverse this situation.

Since all ALK inhibitors exhibit different levels of hepatotoxicity (Ahn et al. 2016), we explored whether the hepatotoxicity of ALK inhibitors was correlated with TXNIP levels. We examined the impact of various ALK inhibitors on TXNIP and discovered a positive correlation between the degree of phosphorylated AKT inhibition and TXNIP upregulation (Fig. S5a), which led us to hypothesize that the hepatotoxicity induced by ALK inhibitors could be driven by the upregulation of TXNIP. To further elucidate the mechanism underlying the ensartinib-induced upregulation of TXNIP transcription, we silenced the transcription factors of TXNIP, namely, Foxo1, MondoA, and ChREBP, which have been previously reported (Li et al. 2015; Xu et al. 2012; Lu et al. 2021). Only silencing MondoA decreased the protein level of TXNIP (Fig. S5b), suggesting that MondoA plays a key role in ensartinib-induced TXNIP upregulation. We also applied two reported TXNIP inhibitors, metformin and verapamil, which can inhibit TXNIP via Foxo1 and ChREBP (Guo et al. 2023; Ismael et al. 2021). The results showed that verapamil did not alter the protein levels of c-PARP or TXNIP (Fig. S5c). Metformin downregulated some TXNIP proteins and only slightly downregulated c-PARP (Fig. S5d). These findings suggested that neither metformin nor verapamil was effective against ensartinib-induced hepatotoxicity. In addition, previous studies have shown that low levels of TXNIP are related to tumorigenesis; therefore, inhibiting the transcription of TXNIP might not be an ideal intervention strategy for ensartinib-induced hepatotoxicity (Sullivan et al. 2018). Therefore, we investigated other methods that could either impair the function of TXNIP or downregulate the protein level of TXNIP.

### Rutin inhibited TXNIP activity and accelerated TXNIP degradation

**Fig. 6:**
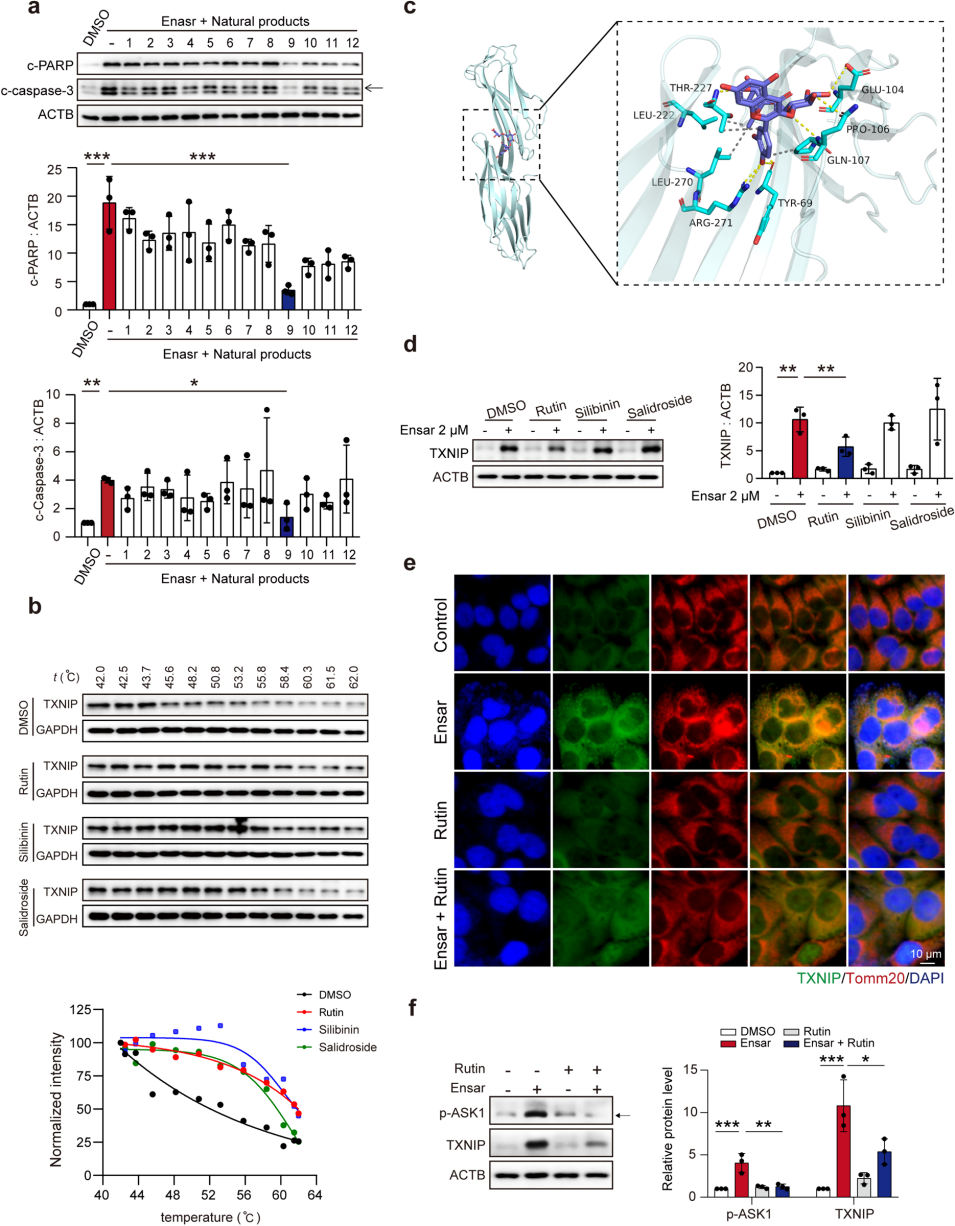
Rutin could inhibit the activity and accelerate the degradation of TXNIP. **(a)** The expression levels of c-PARP and c-caspase-3 in HL-7702 cells treated with 2 μM ensartinib or/and 12 natural compounds for 36 h. **(b)** The binding stability determined by CETSA assay of drug molecules to proteins. **(c)** Molecular docking simulations show that rutin binds to the TXNIP pocket. **(d)** The expression level of TXNIP in HL-7702 cells treated with 2 μM ensartinib or/and 5 μM rutin, silibinin, salidroside for 36 h. **(e)** The localization of TXNIP (original magnification: 400×) was observed by immunofluorescence. Scale bar: 10 μm. **(f)** The expression levels of TXNIP and p-ASK1 in HL-7702 cells treated with 2 μM ensartinib or/and 5 μM rutin (*n* = 3). Data were expressed as mean ± SD. *P < 0.05, **P < 0.01, ***P < 0.001. One way ANOVA followed by Tukey post hoc test for **(a)**, **(d)** and **(f)**. Ensar, ensartinib

Previous research using CB-dock identified 12 natural compounds that could bind and inhibit the activity of TXNIP (Table S1) (Zhang et al. 2021a). We evaluated the ability of these compounds to reverse the ensartinib-induced increase in c-PARP and c-caspase-3 levels. The results demonstrated that rutin, silibinin, and salidroside were the three most effective compounds, with rutin showing the strongest effect (Fig. 6a). To further investigate the interaction between these compounds and TXNIP, we employed cellular thermal shift assay (CETSA), a technique that facilitates the study of drug candidate target engagement within a cellular context (Jafari et al. 2014). Our findings confirmed that all three compounds, rutin, silibinin, and salidroside, could robustly interact with TXNIP (Fig. 6b). The docking of rutin with TXNIP demonstrated that rutin could bind to amino acid residues 69, 104, 106, 107, 222, 227, 270 and 271 of TXNIP (Fig. 6c). Moreover, rutin decreased the protein level of TXNIP induced by ensartinib but did not affect its transcription (Fig. 6d, e, Fig. S6a). We further tested whether the proteasomal inhibitor MG-132, not the autophagy inhibitor chloroquine (CQ), could reverse the downregulation of TXNIP and found that rutin promotes the degradation of TXNIP through the proteasomal pathway (Fig. S6b).

Typically, TXNIP is found in the nucleus under normal conditions, allowing mitochondrion-localized TRX2 to bind to ASK1, thereby inhibiting ASK1 activation and promoting cell survival. However, oxidative stress triggers TXNIP relocation to mitochondria. Here, TXNIP formed a TXNIP/TRX2 complex by competing with ASK1 and thus leading to the phosphorylation of ASK1. This sequence of events stimulated the release of cytochrome c into the cytosol, activating the apoptosis cascade (Choi and Park 2023). Upon combination treatment with rutin, the increased colocalization between TXNIP and mitochondrial was significantly decreased, indicating a robust inhibitory effect on TXNIP activity (Fig. 6e). Moreover, rutin inhibited ASK1 phosphorylation, suggesting its potential to ameliorate the damage induced by TXNIP (Fig. 6f). These findings demonstrated that rutin affects the cellular effect of TXNIP in two ways: on the one hand, rutin inhibits TXNIP activity, while on the other hand, rutin can also promote TXNIP degradation, thereby inhibiting ensartinib-related hepatotoxicity.

### Rutin could ameliorate ensartinib-induced hepatotoxicity *in vitro*

**Fig. 7:**
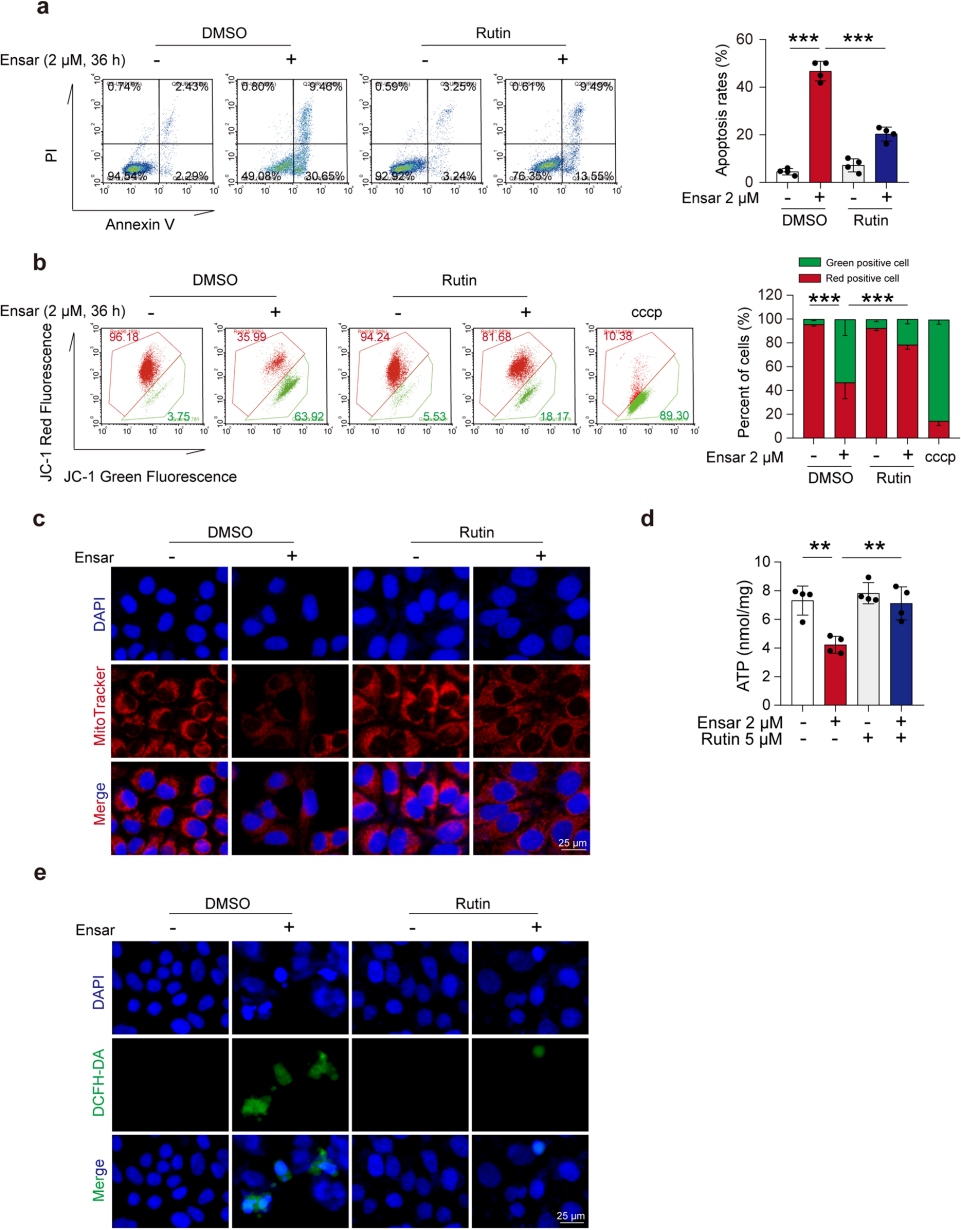
Rutin attenuated hepatocyte apoptosis and mitochondrial damage induced by ensartinib. **(a-e)** HL-7702 cells were treated with 2 μM ensartinib or/and 5 μM rutin for 36 h. **(a)** The apoptosis rates of HL-7702 cells (*n* = 4). **(b)** MMP detected by flow cytometry with JC-1 staining of HL-7702 cells (*n* = 3). cccp was used as a positive control. **(c)** Mitochondrial mass (original magnification: 400×) was detected by MitoTracker staining (red). Scale bar: 25 μm. **(d)** The ATP levels in HL-7702 cells (*n* = 4). **(e)** The ROS level (original magnification: 400×) was observed by DCFH-DA staining. Scale bar: 25 μm. Data were expressed as mean ± SD. **P < 0.01, ***P < 0.001. One way ANOVA followed by Tukey post hoc test for **(a)**, **(b)** and **(d)**. Ensar, ensartinib

We treated HL-7702 cells with ensartinib and/or rutin and subsequently assessed cell viability. Compared to treatment with ensartinib alone, additional rutin treatment significantly improved the survival fraction and morphology of HL-7702 cells (Fig. S7a, b), indicating that rutin can intervene in ensartinib-induced hepatotoxicity. The percentage of apoptotic cells and the degree of mitochondrial damage were significantly reversed after the combined use of rutin and ensartinib than after treatment with ensartinib alone (Fig. 7a, b). Western blot results showed that rutin also reversed the changes in the protein levels of c-PARP and c-caspase-3 induced by ensartinib (Fig. S7c). The protective effect of rutin on mitochondria was further demonstrated by MitoTracker and ATP assays (Fig. 7c, d). Moreover, the addition of rutin alleviated the increase in cellular ROS levels caused by ensartinib treatment (Fig. 7e). Taken together, these findings indicate that rutin could ameliorate the damage caused by ensartinib at the cellular level.

### Rutin could ameliorate ensartinib-induced hepatotoxicity *in vivo*

**Fig. 8:**
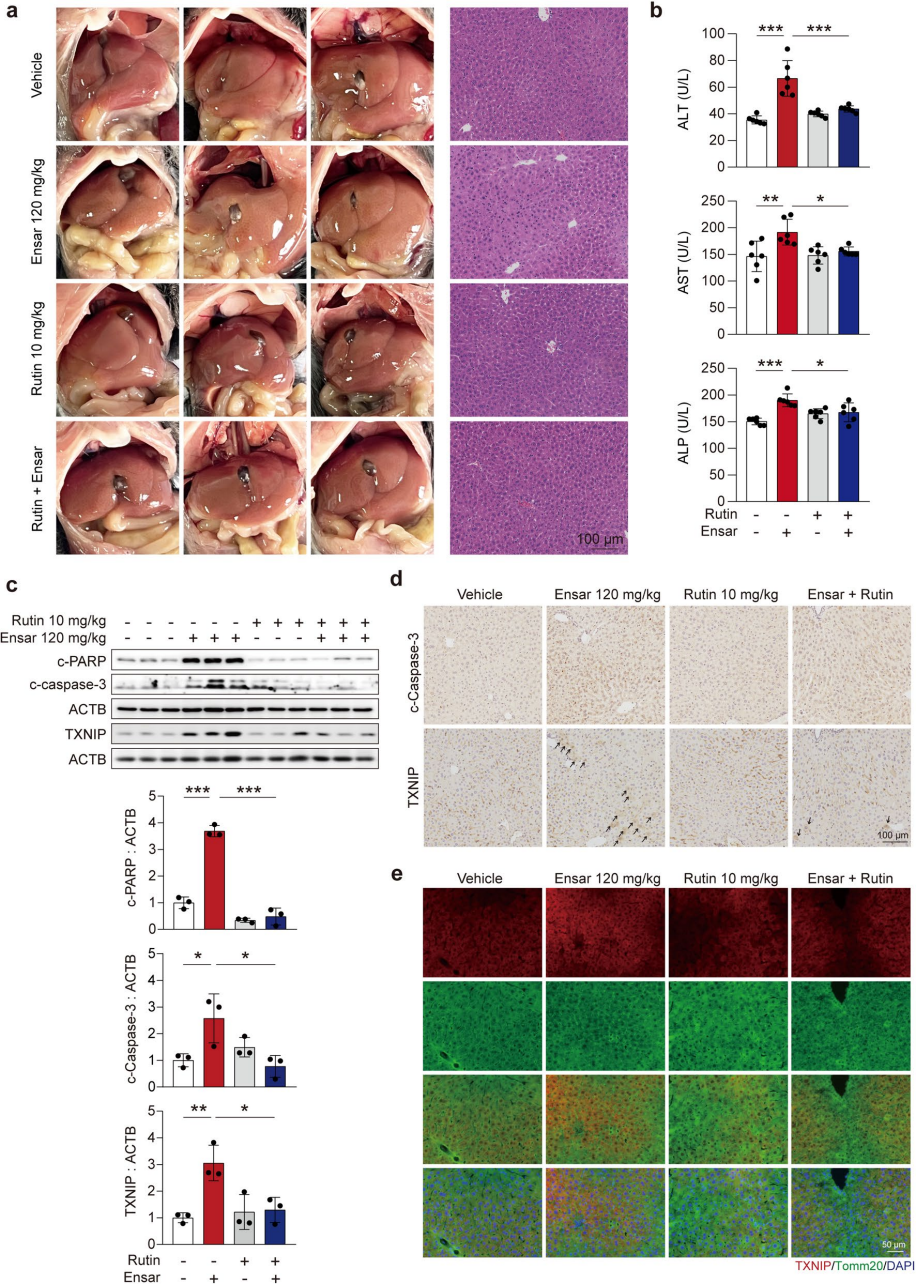
Rutin attenuated liver injury induced by ensartinib in mice. **(a-e)** C57BL/6J male mice were treated with 120 mg/kg/day ensartinib and/or 10 mg/kg/day rutin for 4 weeks (*n* = 6). **(a)** Representative images of liver and representative images of H&E staining (original magnification: 200×). Scale bar: 100 μm. **(b)** The levels of serum ALT, AST and ALP. **(c)** The expression levels of c-PARP, c-caspase-3 and TXNIP in liver tissues. **(d)** Representative images (original magnification: 200×) of immunohistochemistry for c-caspase-3 and TXNIP staining in liver tissues. Scale bar: 100 μm. **(e)** The localization of TXNIP (original magnification: 200×) was observed by immunofluorescence. Scale bar: 50 μm. Data were expressed as mean ± SD. *P < 0.05, **P < 0.01, ***P < 0.001. One way ANOVA followed by Tukey post hoc test for **(b)** and **(c)**. Ensar, ensartinib

Based on these previous findings, we decided to examine the protective role of rutin *in vivo*. C57BL/6J mice were treated with 10 mg/kg rutin with or without 120 mg/kg ensartinib for 4 weeks, and serum and liver samples were collected at the end of the administration period. *In vivo* and H&E staining showed that rutin markedly improved the abnormalities induced by ensartinib treatment (Fig. 8a). Additionally, the serum levels of ALT, AST, and ALP were markedly lower when rutin was combined with ensartinib than when ensartinib was used alone, further underscoring the liver-protective effect of rutin (Fig. 8b). Moreover, Masson’s trichrome and Sirius red staining also indicated that rutin improved the liver fibrosis induced by ensartinib treatment (Fig. S8a, b). The protein levels of c-PARP, c-caspase-3, and TXNIP noticeably decreased after combination treatment with rutin and ensartinib compared with ensartinib treatment alone (Fig. 8c). Immunohistochemistry analysis further confirmed that the abnormal expression of c-caspase-3 and TXNIP triggered by ensartinib administration could be mitigated by rutin (Fig. 8d). Furthermore, the results demonstrated that rutin downregulated not only the protein level of TXNIP but also the colocalization of TXNIP with mitochondria (Fig. 8e).

To validate the impact of rutin on the anticancer activity of ensartinib, we assessed the effect of combining rutin with ensartinib on the ALK-sensitive lung cancer cell line NCI-H3122. The results suggested that rutin did not attenuate the antitumor effect of ensartinib, while rutin itself could partially decrease the viability of NCI-H3122 cells. This finding indicates that rutin is a potentially clinically safe and feasible therapeutic strategy (Fig. S9).

## Discussion

In this study, we demonstrated that mitochondrial dysfunction-mediated hepatocyte apoptosis contributes to ensartinib-induced hepatotoxicity. We showed that the transcriptional activation and mitochondrial translocation of TXNIP are associated with mitochondrial membrane potential loss, ROS accumulation and cell apoptosis. By altering the level or activity of TXNIP, ensartinib-induced hepatocyte apoptosis and related phenotypes can be alleviated. We also investigated several predicted TXNIP inhibitors, especially natural active compounds. Rutin, the strongest inhibitor, can bind and slightly regulate the stability of TXNIP and has the ability to protect against ensartinib-induced hepatocyte apoptosis.

Hepatotoxicity is one of the most common adverse reactions in patients treated with ALK-TKIs, and those treated with crizotinib, ceritinib and ensartinib are at high risk for hepatotoxicity (Zhou et al. 2023). PI3K-AKT is downstream of several tyrosine receptors and negatively regulates the transcription of TXNIP (Zhang et al. 2021b). In our study, we treated hepatocytes with approved ALK-TKIs at a 2-fold Cmax. The results showed that TXNIP increased with AKT inhibition and was correlated with phenotype, especially in the crizotinib- and ensartinib-treated groups. Although ceritinib strongly impaired hepatocytes, TXNIP was less affected by the weak inhibition of AKT. The mechanism involved in ceritinib-induced hepatotoxicity may be unique. The location, function and binding substrate should be simultaneously considered because the level of TXNIP cannot directly reflect cell damage.

TXNIP is a multifunctional protein that pathophysiologically regulates cardiovascular diseases (Wang and Yoshioka 2017) and diabetes (Alhawiti et al. 2017). Suppression of TXNIP seems to be a promising strategy, while completely blocking the activity of TXNIP may cause disadvantageous effects, such as enhanced susceptibility to cancer and deteriorating the fasting response. As reported, TNXIP is a candidate tumor suppressor gene, and TXNIP-deficient mice are predisposed to hepatocellular carcinoma (Sheth et al. 2006). Therefore, increasing TXNIP or inhibiting the specific functional route would be a more precise strategy. To date, most TXNIP inhibitors have been reported to suppress the expression of TXNIP by affecting transcription factors. SBI-477 and SBI-993 function as insulin signaling inducers by deactivating the transcription factor MondoA and thus reducing the expression of TXNIP (Ahn et al. 2016). Verapamil and metformin not only decreased the nuclear entry of ChREBP and/or FOXO1 but also reduced its recruitment to the E-box repeat in the TXNIP promoter, which resulted in TXNIP transcriptional inhibition (Li et al. 2015). In our study, we found that, rather than ChREBP or FOXO1, ensartinib increases the level of TXNIP in a MondoA regulated manner. Although metformin can slightly decrease the level of TXNIP, this effect may be related to the activation of AMPK, which is reported to directly phosphorylate TXNIP at Ser 308 and accelerate its degradation in response to energy stress (Wu et al. 2013). Other methods to regulate TXNIP expression at the protein level have also been reported. The E3 ligase ITCH targets TXNIP for polyubiquitination and induces TXNIP to undergo prosomal degradation under basal conditions (Zhang et al. 2010), while the deubiquitinase USP5 can stabilize TNXIP under lipopolysaccharide treatment (Shi et al. 2023). Therefore, developing selective chemicals targeting TNXIP degradation would also be feasible given that some reported inhibitors have achieved promising results. Regarding ubiquitination inhibitors or agonists, the selectivity of the target of interest and the effect of dyshomeostasis on other substrates should be considered. As TXNIP functions through shuttling and binding with other substrates, hindering specific motifs not only serves as a method for designing novel inhibitors but also deepens the understanding of signal transduction mechanisms from another aspect. The peptide TN13, which mimics the interaction motif of TXNIP-p38, substantially interferes with the function of p38 and reactivates aged hematopoietic stem cells (Jung et al. 2016). Whether small molecules can inhibit TXNIP activity by blocking specific sections remains elusive.

Rutin, a clinically feasible supplement, is used to reduce capillary permeability and prevent hypertensive cerebral hemorrhage, diabetic retinal hemorrhage and hemorrhagic purpura. Due to its anti-inflammatory and antioxidant effects, rutin has beneficial biological functions in several diseases, including diabetes (Ghorbani 2017), neurodegenerative diseases (Enogieru et al. 2018), and autoimmune diseases (Nikfarjam et al. 2017). The antioxidant effect of rutin is associated with inducing the NRF2/ARE pathway and enhancing the expression of enzymes involved in redox regulation (Rahmani et al. 2023), while rutin can directly scavenge ROS through its chemical structure (Hanasaki et al. 1994). Hydrolyzed rutin-derived quercetin has also been reported to negatively regulate the activity of xanthine oxidase, which is involved in the production of ROS (Tang et al. 2019); however, the potential target of rutin itself has been less investigated. In this study, rutin was the most effective reactive compound for both binding TXNIP and alleviating ensartinib-induced apoptosis. Interestingly, rutin slightly decreased the expression level of TXNIP compared with those of silibinin and salidroside, while the transcript level did not significantly change, which may be the reason that among several phytochemicals, rutin has emerged as the best candidate. Apart from the known mechanisms of existing TXNIP inhibitors, rutin seems to play a novel role in regulating downstream TXNIP, and concrete details of this mechanism merit further investigation. TXNIP has an intramolecular disulfide bond between Cys63 and Cys247, which is essential for disulfide exchange with reduced TRX (Jiang et al. 2022). We found that rutin could bind to amino acid residue 69 of TXNIP and may influence disulfide bond switching, thereby inhibiting ASK1 activation and apoptosis. Additionally, the outcome of rutin binding may affect the interaction between TXNIP and other substrates and the stability of its ability to slightly accelerate its proteasomal degradation. We used CETSA and molecular docking to confirm the binding between rutin and TXNIP. Further studies concerning direct evidence, such as small molecule labeling and pull-down, fluorescence polarization immunoassays and microscale thermophoresis, should be carried out. We also used the ALK-sensitive lung cancer cell line NCI-H3122 to test the signaling pathway and function of rutin. Rutin did not attenuate the antitumor effect of ensartinib, while rutin itself partially decreased the viability of NCI-H3122 cells. Rutin has been reported to modulate several signaling pathways to exert its anticancer potential and has a synergistic effect on inducing apoptosis when combined with therapeutic agents (Satari et al. 2021). In cancer, the regulation of TXNIP may be one of the mechanisms underlying proliferation and cancer progression, and TXNIP has a relatively small effect on tumor survival compared to the overall pathway-mediated inhibition of oncogenic drivers. Rutin tablets and troxerutin tablets are currently available in clinical practice and show high safety, and combination strategies could be used in a preliminary clinical trial. More details about the effect of rutin on tumor progression and the feasibility of its new clinical use need to be fully considered.

**Fig. 9:**
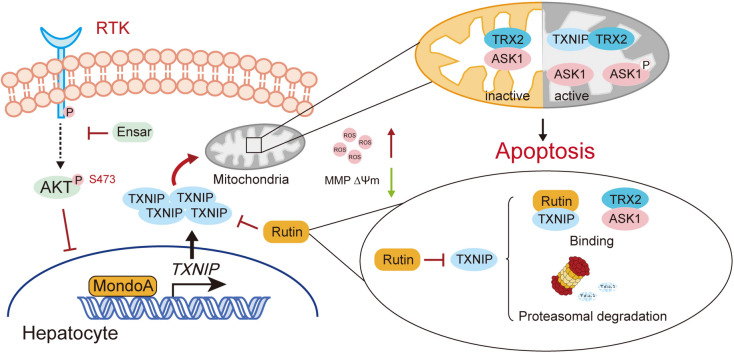
Schematic diagram of rutin protects liver from ensartinib-induced hepatotoxicity. Ensartinib increases TXNIP by transcriptional activation which is related to AKT inhibition and is mediated by MondoA. A high level of TXNIP translocates to mitochondria, activate ASK1 and leads to hepatocyte mitochondrial-dependent apoptosis. Rutin could inhibit TXNIP by binding to TXNIP and partially promoting its proteasomal degradation and possess a favorable effect for intervening ensartinib-induced hepatoxicity

Taken together, our data reveal that the hepatotoxicity of ensartinib is dependent on MondoA-mediated transcriptional activation of TXNIP. Rutin could effectively inhibit activation of TXNIP-mediated apoptosis, and the mechanism might be related to structural modification-induced functional changes and protein instability in TXNIP rather than transcriptional inhibition, as reported for TXNIP inhibitors (Fig. 9). In addition to having a protective effect on ensartinib-induced hepatotoxicity, rutin could also slightly strengthen its antitumor activity. This research provides insight into the mechanism of kinase inhibitor-induced hepatotoxicity and the function of TXNIP in regulating hepatocyte homeostasis.

## Supplementary information

ESM 1 (DOCX 93950 kb)

## Data Availability

Supporting data for the findings in this paper can be obtained upon request from the corresponding authors. The TXNIP crystal structure (PDB ID 4II1) was sourced from the protein data bank, while the molecular structure of rutin (PubChem CID 5280805) was retrieved from the PubChem database. Additionally, the RNA-seq data have been made available in the NCBI Gene Expression Omnibus database, accessible under the accession number GSE249370.

## References

[CR1] Ahn B, Soundarapandian MM, Sessions H, Peddibhotla S, Roth GP, Li JL, Sugarman E, Koo A, Malany S, Wang M, et al. MondoA coordinately regulates skeletal myocyte lipid homeostasis and insulin signaling. J Clin Invest. 2016;126(9):3567–79. 10.1172/jci87382. 27500491 10.1172/JCI87382 PMC5004938

[CR2] Ai X, Wang Q, Cheng Y, Liu X, Cao L, Chen J, Dong X, Zhou J, Fan Y, Huang C, et al. Safety but limited efficacy of ensartinib in ROS1-positive NSCLC: a single-arm, multicenter phase 2 study. J Thorac Oncol. 2021;16(11):1959–63. 10.1016/j.jtho.2021.06.023. 34265433 10.1016/j.jtho.2021.06.023

[CR3] Alhawiti NM, Al Mahri S, Aziz MA, Malik SS, Mohammad S. TXNIP in metabolic regulation: physiological role and therapeutic outlook. Curr Drug Targets. 2017;18(9):1095–103. 10.2174/1389450118666170130145514. 28137209 10.2174/1389450118666170130145514 PMC5543564

[CR4] Andrade RJ, Chalasani N, Bjornsson ES, Suzuki A, Kullak-Ublick GA, Watkins PB, Devarbhavi H, Merz M, Lucena MI, Kaplowitz N, et al. Drug-induced liver injury. Nat Rev Dis Primers. 2019;5(1):58. 10.1038/s41572-019-0105-0. 31439850 10.1038/s41572-019-0105-0

[CR5] Ao H, Li H, Zhao X, Liu B, Lu L. TXNIP positively regulates the autophagy and apoptosis in the rat müller cell of diabetic retinopathy. Life Sci. 2021;267:118988. 10.1016/j.lfs.2020.118988. 33412212 10.1016/j.lfs.2020.118988

[CR6] Bock FJ, Tait SWG. Mitochondria as multifaceted regulators of cell death. Nat Rev Mol Cell Biol. 2020;21(2):85–100. 10.1038/s41580-019-0173-8. 31636403 10.1038/s41580-019-0173-8

[CR7] Chen J, Saxena G, Mungrue IN, Lusis AJ, Shalev A. Thioredoxin-interacting protein: a critical link between glucose toxicity and beta-cell apoptosis. Diabetes. 2008;57(4):938–44. 10.2337/db07-0715. 18171713 10.2337/db07-0715 PMC3618659

[CR8] Choi EH, Park SJ. TXNIP: a key protein in the cellular stress response pathway and a potential therapeutic target. Exp Mol Med. 2023;55(7):1348–56. 10.1038/s12276-023-01019-8. 37394581 10.1038/s12276-023-01019-8 PMC10393958

[CR9] Enogieru AB, Haylett W, Hiss DC, Bardien S, Ekpo OE. Rutin as a potent antioxidant: implications for neurodegenerative disorders. Oxidative Med Cell Longev. 2018;2018:6241017. 10.1155/2018/6241017. 10.1155/2018/6241017 PMC6040293 30050657

[CR10] Ferreira LG, Dos Santos RN, Oliva G, Andricopulo AD. Molecular docking and structure-based drug design strategies. Molecules. 2015;20(7):13384–421. 10.3390/molecules200713384. 26205061 10.3390/molecules200713384 PMC6332083

[CR11] Fukui T, Tachihara M, Nagano T, Kobayashi K. Review of therapeutic strategies for anaplastic lymphoma kinase-rearranged non-small cell lung cancer. Cancers (Basel). 2022;14(5) 10.3390/cancers14051184. 10.3390/cancers14051184 PMC8909087 35267492

[CR12] Ghorbani A. Mechanisms of antidiabetic effects of flavonoid rutin. Biomed Pharmacother. 2017;96:305–12. 10.1016/j.biopha.2017.10.001. 29017142 10.1016/j.biopha.2017.10.001

[CR13] Guo H, Fang T, Cheng Y, Li T, Qu JR, Xu CF, Deng XQ, Sun B, Chen LM. ChREBP-β/TXNIP aggravates frucose-induced renal injury through triggering ferroptosis of renal tubular epithelial cells. Free Radic Biol Med. 2023;199:154–65. 10.1016/j.freeradbiomed.2023.02.013. 36828294 10.1016/j.freeradbiomed.2023.02.013

[CR14] Han Y, Xu X, Tang C, Gao P, Chen X, Xiong X, Yang M, Yang S, Zhu X, Yuan S, et al. Reactive oxygen species promote tubular injury in diabetic nephropathy: the role of the mitochondrial ros-txnip-nlrp3 biological axis. Redox Biol. 2018;16:32–46. 10.1016/j.redox.2018.02.013. 29475133 10.1016/j.redox.2018.02.013 PMC5842313

[CR15] Hanasaki Y, Ogawa S, Fukui S. The correlation between active oxygens scavenging and antioxidative effects of flavonoids. Free Radic Biol Med. 1994;16(6):845–50. 10.1016/0891-5849(94)90202-x. 8070690 10.1016/0891-5849(94)90202-x

[CR16] Horn L, Infante JR, Reckamp KL, Blumenschein GR, Leal TA, Waqar SN, Gitlitz BJ, Sanborn RE, Whisenant JG, Du L, et al. Ensartinib (X-396) in ALK-positive non-small cell lung cancer: results from a first-in-human phase I/II, multicenter study. Clin Cancer Res. 2018;24(12):2771–9. 10.1158/1078-0432.Ccr-17-2398. 29563138 10.1158/1078-0432.CCR-17-2398 PMC6004248

[CR17] Horn L, Wang Z, Wu G, Poddubskaya E, Mok T, Reck M, Wakelee H, Chiappori AA, Lee DH, Breder V, et al. Ensartinib vs Crizotinib for patients with anaplastic lymphoma kinase-positive non-small cell lung cancer: a randomized clinical trial. JAMA Oncol. 2021;7(11):1617–25. 10.1001/jamaoncol.2021.3523. 34473194 10.1001/jamaoncol.2021.3523 PMC8414368

[CR18] Iorga A, Dara L, Kaplowitz N. Drug-induced liver injury: cascade of events leading to cell death, apoptosis or necrosis. Int J Mol Sci. 2017;18(5) 10.3390/ijms18051018. 10.3390/ijms18051018 PMC5454931 28486401

[CR19] Ismael S, Nasoohi S, Yoo A, Mirzahosseini G, Ahmed HA, Ishrat T. Verapamil as an adjunct therapy to reduce tPA toxicity in hyperglycemic stroke: implication of TXNIP/NLRP3 inflammasome. Mol Neurobiol. 2021;58(8):3792–804. 10.1007/s12035-021-02384-z. 33847912 10.1007/s12035-021-02384-z PMC8282727

[CR20] Jafari R, Almqvist H, Axelsson H, Ignatushchenko M, Lundbäck T, Nordlund P, Martinez Molina D. The cellular thermal shift assay for evaluating drug target interactions in cells. Nat Protoc. 2014;9(9):2100–22. 10.1038/nprot.2014.138. 25101824 10.1038/nprot.2014.138

[CR21] Jiang N, Liu J, Guan C, Ma C, An J, Tang X. Thioredoxin-interacting protein: A new therapeutic target in bone metabolism disorders? Front Immunol. 2022;13:955128. 10.3389/fimmu.2022.955128. 36059548 10.3389/fimmu.2022.955128 PMC9428757

[CR22] Jung H, Kim DO, Byun JE, Kim WS, Kim MJ, Song HY, Kim YK, Kang DK, Park YJ, Kim TD, et al. Thioredoxin-interacting protein regulates haematopoietic stem cell ageing and rejuvenation by inhibiting p38 kinase activity. Nat Commun. 2016;7:13674. 10.1038/ncomms13674. 27929088 10.1038/ncomms13674 PMC5155146

[CR23] Li X, Kover KL, Heruth DP, Watkins DJ, Moore WV, Jackson K, Zang M, Clements MA, Yan Y. New insight into metformin action: regulation of ChREBP and FOXO1 activities in endothelial cells. Mol Endocrinol. 2015;29(8):1184–94. 10.1210/me.2015-1090. 26147751 10.1210/ME.2015-1090 PMC5414702

[CR24] Lu Y, Li Y, Liu Q, Tian N, Du P, Zhu F, Han Y, Liu X, Liu X, Peng X, et al. MondoA-thioredoxin-interacting protein axis maintains regulatory T-cell identity and function in colorectal cancer microenvironment. Gastroenterology. 2021;161(2):575–591.e516. 10.1053/j.gastro.2021.04.041. 33901495 10.1053/j.gastro.2021.04.041

[CR25] Luo Y, Zhang Z, Guo X, Tang X, Li S, Gong G, Gao S, Zhang Y, Lin S. Comparative safety of anaplastic lymphoma kinase tyrosine kinase inhibitors in advanced anaplastic lymphoma kinase-mutated non-small cell lung cancer: systematic review and network meta-analysis. Lung Cancer. 2023;184:107319. 10.1016/j.lungcan.2023.107319. 37597303 10.1016/j.lungcan.2023.107319

[CR26] Ma Y, Pan H, Liu Y, Zhang Y, Hong S, Huang J, Weng S, Yang Y, Fang W, Huang Y, et al. Ensartinib in advanced ALK-positive non-small cell lung cancer: a multicenter, open-label, two-staged, phase 1 trial. J Thorac Dis. 2022;14(12):4751–62. 10.21037/jtd-22-1606. 36647478 10.21037/jtd-22-1606 PMC9840022

[CR27] Minn AH, Hafele C, Shalev A. Thioredoxin-interacting protein is stimulated by glucose through a carbohydrate response element and induces beta-cell apoptosis. Endocrinology. 2005;146(5):2397–405. 10.1210/en.2004-1378. 15705778 10.1210/en.2004-1378

[CR28] Nikfarjam BA, Adineh M, Hajiali F, Nassiri-Asl M. Treatment with rutin - a therapeutic strategy for neutrophil-mediated inflammatory and autoimmune diseases: - anti-inflammatory effects of rutin on neutrophils. Aust J Pharm. 2017;20(1):52–6. 10.3831/kpi.2017.20.003. 10.3831/KPI.2017.20.003 PMC5374339 28392963

[CR29] Rahmani S, Naraki K, Roohbakhsh A, Hayes AW, Karimi G. The protective effects of rutin on the liver, kidneys, and heart by counteracting organ toxicity caused by synthetic and natural compounds. Food Sci Nutr. 2023;11(1):39–56. 10.1002/fsn3.3041. 36655104 10.1002/fsn3.3041 PMC9834893

[CR30] Satari A, Ghasemi S, Habtemariam S, Asgharian S, Lorigooini Z. Rutin: a flavonoid as an effective sensitizer for anticancer therapy, Insights into Multifaceted Mechanisms and Applicability for Combination Therapy. Evid Based Complement Alternat Med. 2021;2021:9913179. 10.1155/2021/9913179. 34484407 10.1155/2021/9913179 PMC8416379

[CR31] Schneider JL, Lin JJ, Shaw AT. ALK-positive lung cancer: a moving target. Nat Can. 2023;4(3):330–43. 10.1038/s43018-023-00515-0. 10.1038/s43018-023-00515-0 PMC10754274 36797503

[CR32] Schulze PC, De Keulenaer GW, Yoshioka J, Kassik KA, Lee RT. Vitamin D3-upregulated protein-1 (VDUP-1) regulates redox-dependent vascular smooth muscle cell proliferation through interaction with thioredoxin. Circ Res. 2002;91(8):689–95. 10.1161/01.res.0000037982.55074.f6. 12386145 10.1161/01.res.0000037982.55074.f6

[CR33] Shait Mohammed MR, Alzahrani F, Hosawi S, Choudhry H, Khan MI. Profiling the effect of targeting wild isocitrate dehydrogenase 1 (IDH1) on the cellular metabolome of leukemic cells. Int J Mol Sci. 2022;23(12) 10.3390/ijms23126653. 10.3390/ijms23126653 PMC9224363 35743098

[CR34] Sheth SS, Bodnar JS, Ghazalpour A, Thipphavong CK, Tsutsumi S, Tward AD, Demant P, Kodama T, Aburatani H, Lusis AJ. Hepatocellular carcinoma in Txnip-deficient mice. Oncogene. 2006;25(25):3528–36. 10.1038/sj.onc.1209394. 16607285 10.1038/sj.onc.1209394

[CR35] Shi S, Pan X, Chen M, Zhang L, Zhang S, Wang X, Shi S, Chen Z, Lin W, Jiang Y. USP5 promotes lipopolysaccharide-induced apoptosis and inflammatory response by stabilizing the TXNIP protein. Hepatol Commun. 2023;7(8) 10.1097/hc9.0000000000000193. 10.1097/HC9.0000000000000193 PMC10553006 37534934

[CR36] Sullivan WJ, Mullen PJ, Schmid EW, Flores A, Momcilovic M, Sharpley MS, Jelinek D, Whiteley AE, Maxwell MB, Wilde BR, et al. Extracellular matrix remodeling regulates glucose metabolism through TXNIP destabilization. Cell. 2018;175(1):117–132.e121. 10.1016/j.cell.2018.08.017. 30197082 10.1016/j.cell.2018.08.017 PMC6151140

[CR37] Tang X, Tang P, Ma L, Liu L. Screening and evaluation of xanthine oxidase inhibitors from Gnetum parvifolium in China. Molecules. 2019;24(14) 10.3390/molecules24142671. 10.3390/molecules24142671 PMC6680845 31340570

[CR38] Velma V, Tchounwou PB. Oxidative stress and DNA damage induced by chromium in liver and kidney of goldfish, carassius auratus. Biomark Insights. 2013;8:43–51. 10.4137/bmi.S11456. 23700361 10.4137/BMI.S11456 PMC3653851

[CR39] Wang BF, Yoshioka J. The emerging role of thioredoxin-interacting protein in myocardial ischemia/reperfusion injury. J Cardiovasc Pharmacol Ther. 2017;22(3):219–29. 10.1177/1074248416675731. 27807222 10.1177/1074248416675731 PMC5389923

[CR40] Wang H, Liu D, Sun Y, Meng C, Tan L, Song C, Qiu X, Liu W, Ding C, Ying L. Upregulation of DUSP6 impairs infectious bronchitis virus replication by negatively regulating ERK pathway and promoting apoptosis. Vet Res. 2021;52(1):7. 10.1186/s13567-020-00866-x. 33431056 10.1186/s13567-020-00866-x PMC7798014

[CR41] Wu N, Zheng B, Shaywitz A, Dagon Y, Tower C, Bellinger G, Shen CH, Wen J, Asara J, McGraw TE, et al. AMPK-dependent degradation of TXNIP upon energy stress leads to enhanced glucose uptake via GLUT1. Mol Cell. 2013;49(6):1167–75. 10.1016/j.molcel.2013.01.035. 23453806 10.1016/j.molcel.2013.01.035 PMC3615143

[CR42] Xu G, Chen J, Jing G, Shalev A. Preventing β-cell loss and diabetes with calcium channel blockers. Diabetes. 2012;61(4):848–56. 10.2337/db11-0955. 22442301 10.2337/db11-0955 PMC3314354

[CR43] Xu Y, Zhou Z, Kang X, Pan L, Liu C, Liang X, Chu J, Dong S, Li Y, Liu Q, et al. Mettl3-mediated mRNA m(6)A modification controls postnatal liver development by modulating the transcription factor Hnf4a. Nat Commun. 2022;13(1):4555. 10.1038/s41467-022-32169-4. 35931692 10.1038/s41467-022-32169-4 PMC9355946

[CR44] Yang Y, Zhou J, Zhou J, Feng J, Zhuang W, Chen J, Zhao J, Zhong W, Zhao Y, Zhang Y, et al. Efficacy, safety, and biomarker analysis of ensartinib in crizotinib-resistant, ALK-positive non-small-cell lung cancer: a multicentre, phase 2 trial. Lancet Respir Med. 2020;8(1):45–53. 10.1016/S2213-2600(19)30252-8. 31628085 10.1016/S2213-2600(19)30252-8

[CR45] Zhang M, Hu G, Shao N, Qin Y, Chen Q, Wang Y, Zhou P, Cai B. Thioredoxin-interacting protein (TXNIP) as a target for alzheimer's disease: flavonoids and phenols. Inflammopharmacology. 2021a;29(5):1317–29. 10.1007/s10787-021-00861-4. 34350508 10.1007/s10787-021-00861-4

[CR46] Zhang P, Wang C, Gao K, Wang D, Mao J, An J, Xu C, Wu D, Yu H, Liu JO, et al. The ubiquitin ligase itch regulates apoptosis by targeting thioredoxin-interacting protein for ubiquitin-dependent degradation. J Biol Chem. 2010;285(12):8869–79. 10.1074/jbc.M109.063321. 20068034 10.1074/jbc.M109.063321 PMC2838308

[CR47] Zhang X, Zhao S, Yuan Q, Zhu L, Li F, Wang H, Kong D, Hao J. TXNIP, a novel key factor to cause Schwann cell dysfunction in diabetic peripheral neuropathy, under the regulation of PI3K/Akt pathway inhibition-induced DNMT1 and DNMT3a overexpression. Cell Death Dis. 2021b;12(7):642. 10.1038/s41419-021-03930-2. 34162834 10.1038/s41419-021-03930-2 PMC8222353

[CR48] Zhang Y, Huang Z, Zeng L, Zhang X, Li Y, Xu Q, Yang H, Lizaso A, Xu C, Liu J, et al. Disease progression patterns and molecular resistance mechanisms to crizotinib of lung adenocarcinoma harboring ROS1 rearrangements. NPJ Precis Oncol. 2022;6(1):20. 10.1038/s41698-022-00264-w. 35361870 10.1038/s41698-022-00264-w PMC8971474

[CR49] Zhou F, Yang Y, Zhang L, Cheng Y, Han B, Lu Y, Wang C, Wang Z, Yang N, Fan Y, et al. Expert consensus of management of adverse drug reactions with anaplastic lymphoma kinase tyrosine kinase inhibitors. ESMO Open. 2023;8(3):101560. 10.1016/j.esmoop.2023.101560. 37230029 10.1016/j.esmoop.2023.101560 PMC10225873

[CR50] Zorov DB, Juhaszova M, Sollott SJ. Mitochondrial reactive oxygen species (ROS) and ROS-induced ROS release. Physiol Rev. 2014;94(3):909–50. 10.1152/physrev.00026.2013. 24987008 10.1152/physrev.00026.2013 PMC4101632

