# Supplementary material for: Rutin attenuates ensartinib-induced hepatotoxicity by non-transcriptional regulation of TXNIP
Source: Cell Biol Toxicol. 2024 May 24;40(1):38. doi: 10.1007/s10565-024-09883-4 (PMC11126486; doi:10.1007/s10565-024-09883-4)
Supplement: Supplementary file 1 — (DOCX 93950 kb) [file 10565_2024_9883_MOESM1_ESM.docx]

**Rutin attenuates ensartinib-induced hepatotoxicity by non-transcriptional regulation of TXNIP**

Wentong Wu^a, 1^, Jinjin Li^a, 1^, Yiming Yin^a^, Yourong Zhou^a^, Xiangliang Huang^a^, Yashi Cao^a^, Xueqin Chen^b,c^, Yunfang Zhou^d^, Jiangxia Du^e^, Zhifei Xu^a^, Bo Yang^f,g^, Qiaojun He^a,g,h^, Xiaochun Yang^a^, Yuhuai Hu^i^, Hao Yan^a*^, Peihua Luo^a,j,k*^

^a^Center for Drug Safety Evaluation and Research of Zhejiang University, College of Pharmaceutical Sciences, Zhejiang University, Hangzhou 310058, China

^b^Department of Oncology, Affiliated Hangzhou Cancer Hospital, Zhejiang University School of Medicine, Key Laboratory of Clinical Cancer Pharmacology and Toxicology Research of Zhejiang Province, Hangzhou 310002, China

^c^Cancer Center, Zhejiang University, Hangzhou 310058, China

^d^The Laboratory of Clinical Pharmacy, the Sixth Affiliated Hospital of Wenzhou Medical University, The People's Hospital of Lishui, Lishui 323020, China

^e^Center for Medical Research and Innovation in Digestive System Tumors, Ministry of Education, the Second Affiliated Hospital, Zhejiang University School of Medicine, Hangzhou 310017, China

^f^Institute of Pharmacology & Toxicology, College of Pharmaceutical Sciences, Zhejiang University, Hangzhou 310058, China

^g^School of Medicine, Hangzhou City University, Hangzhou 310015, China

^h^Innovation Institute for Artificial Intelligence in Medicine of Zhejiang University, Hangzhou 310018, China

^i^Innovation Institute of Hangzhou Yuhong Pharmatech Co.,LTD, Hangzhou 310018, China.

^j^Department of Pharmacology and Toxicology, Hangzhou Institute of Innovative Medicine, College of Pharmaceutical Sciences, Zhejiang University, Hangzhou 310018, China.

^k^Key Laboratory of Clinical Cancer Pharmacology and Toxicology Research of Zhejiang Province, Affiliated Hangzhou Cancer Hospital, Zhejiang University School of Medicine, Hangzhou 310002, China.

^*^Corresponding authors at**:** 866 Yuhangtang Road, Zijingang Campus, Zhejiang University, Hangzhou 310058, Zhejiang, China

*Email addresses*: yh925@zju.edu.cn (H. Yan), peihualuo@zju.edu.cn (P. Luo)

^1^These authors contributed to this work equally.

**Supplemental information**


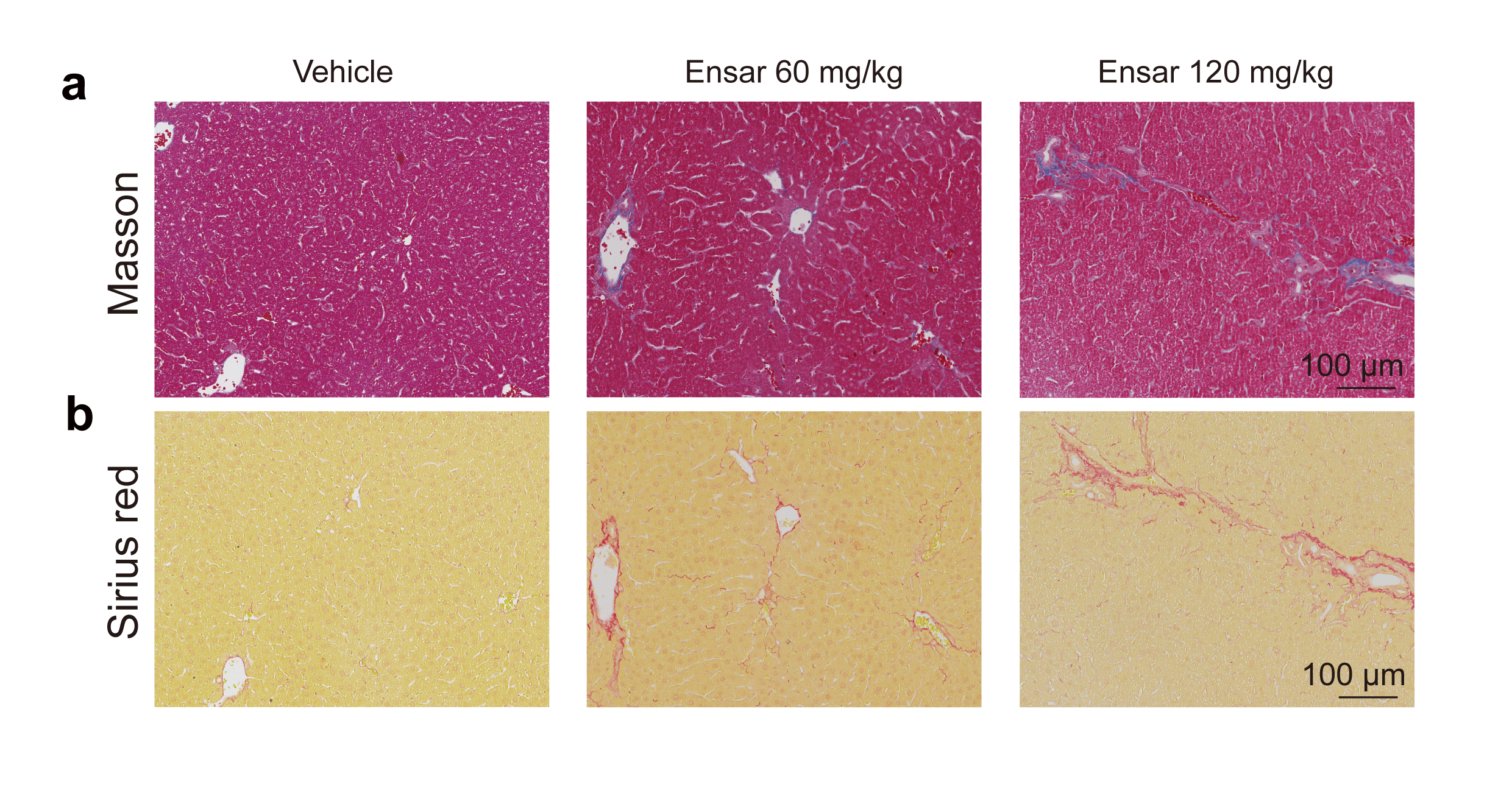


**Fig. S1 Ensartinib induces liver fibrosis *in vivo*. (a-b)** C57BL/6J male mice were treated with 0.5% CMC-Na, 60 mg/kg/day or 120 mg/kg/day ensartinib for 4 weeks. Representative images (original magnification: 200×) of Masson’s staining **(a)** and Sirius Red staining **(b)** in liver tissues from C57BL/6J mice. Scale bar: 100 µm. Ensar, ensartinib.


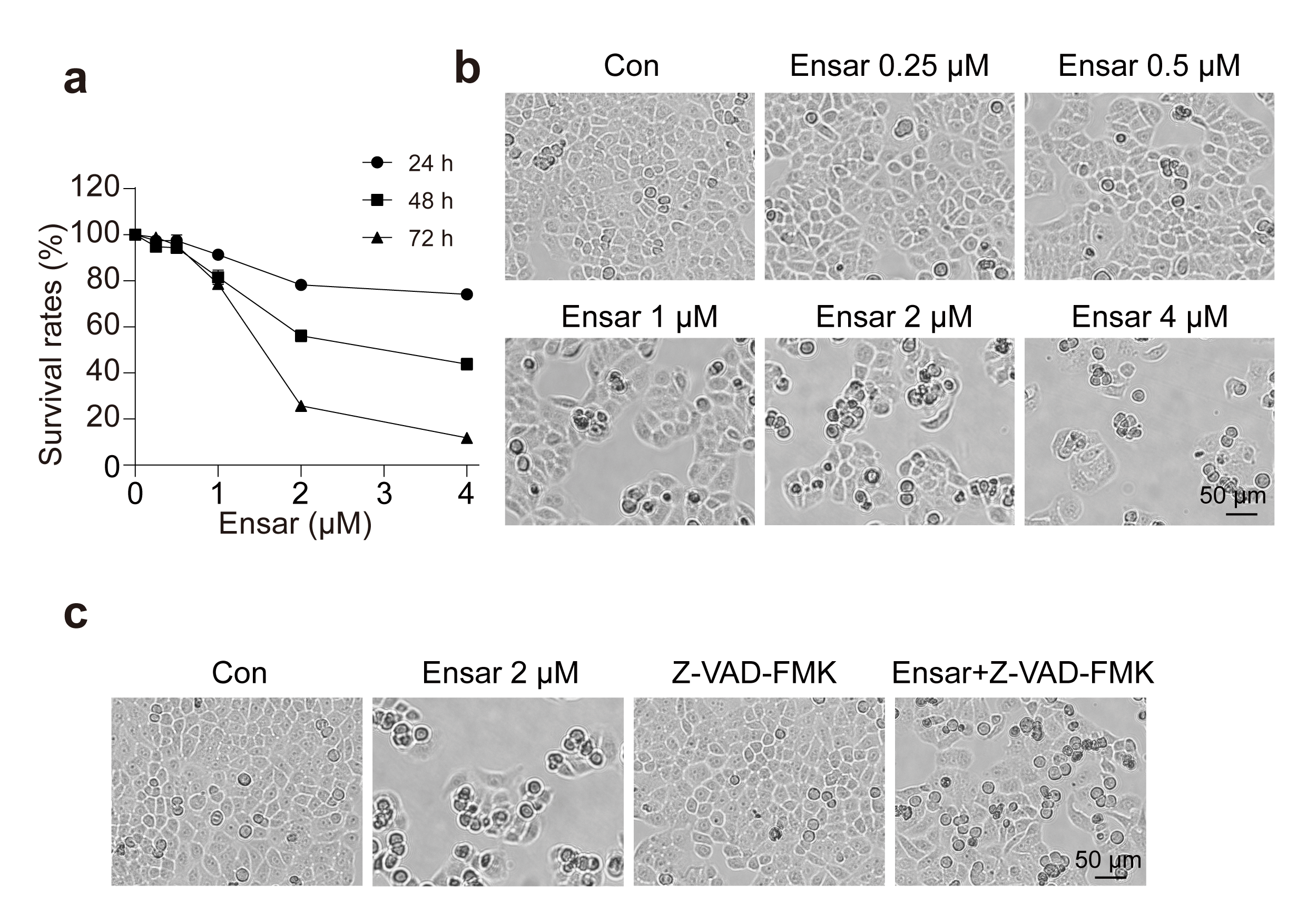


**Fig. S2 Ensartinib induces hepatocyte apoptosis. (a)** The survival rates of HL-7702 cells treated with 0, 0.25, 0.5, 1, 2 or 4 μM ensartinib for 24 h, 48 h or 72 h were detected by SRB staining. n = 3 independent experiments. **(b)** The representative images (original magnification: 200×) of HL-7702 cells treated were taken with a normal light microscope. Scale bar: 50 µm. **(c)** The representative images (original magnification: 200×) of HL-7702 cells treated with 2 μM ensartinib and/or 20 µM Z-VAD-FMK were taken with a normal light microscope. Scale bar: 50 µm. Ensar, ensartinib.


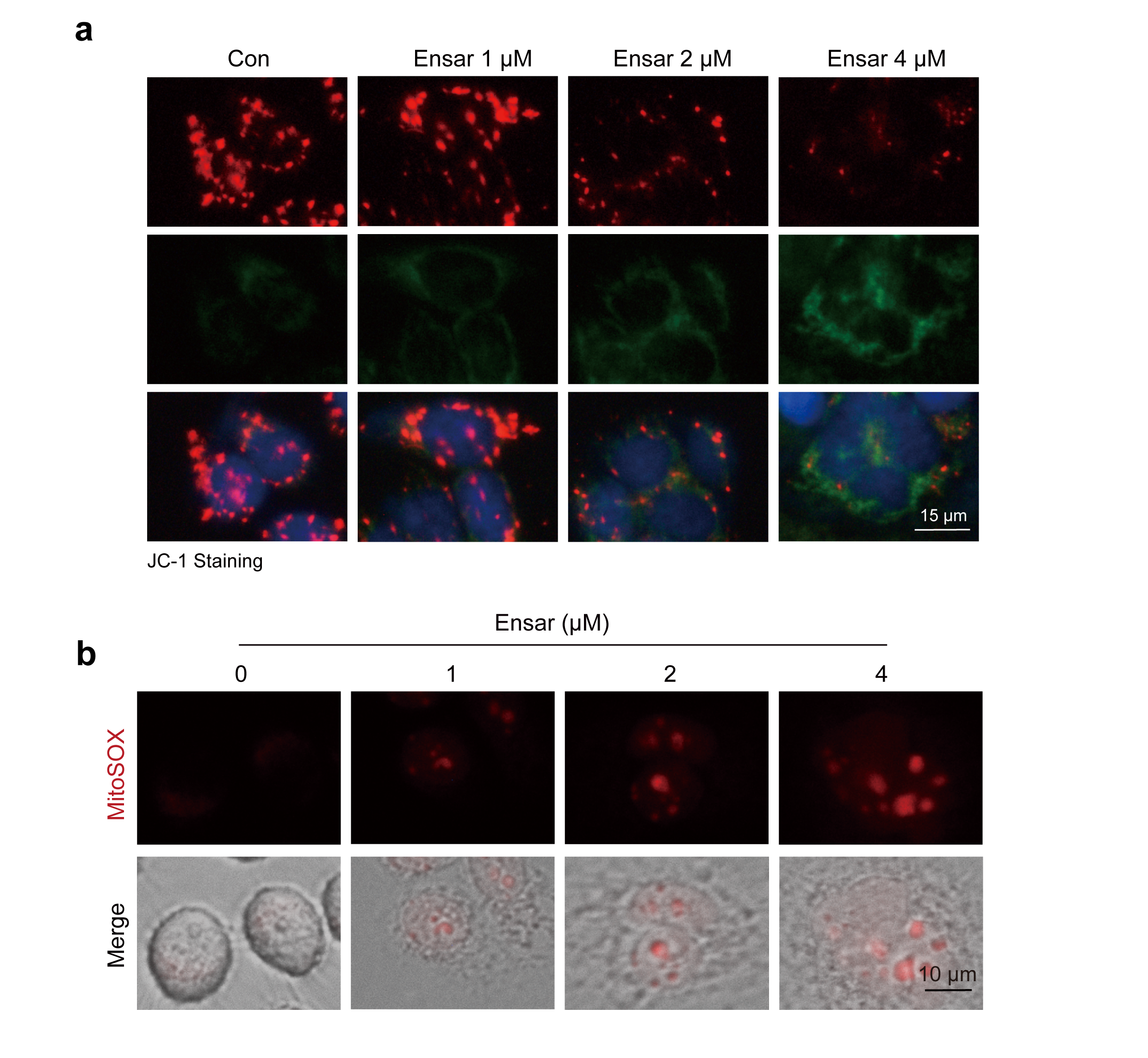


**Fig. S3 Ensartinib causes mitochondrial damage in hepatocytes. (a-b)** HL-7702 cells were treated with 0, 1, 2 or 4 μM ensartinib for 36 h. **(a)** Representative images (original magnification: 400×) of liver of JC-1 staining. Scale bar: 15 µm. **(b)** Mitochondrial ROS was detected by MitoSOX staining (red). Representative images (original magnification: 400×) are shown. Scale bar: 10 µm. Ensar, ensartinib.


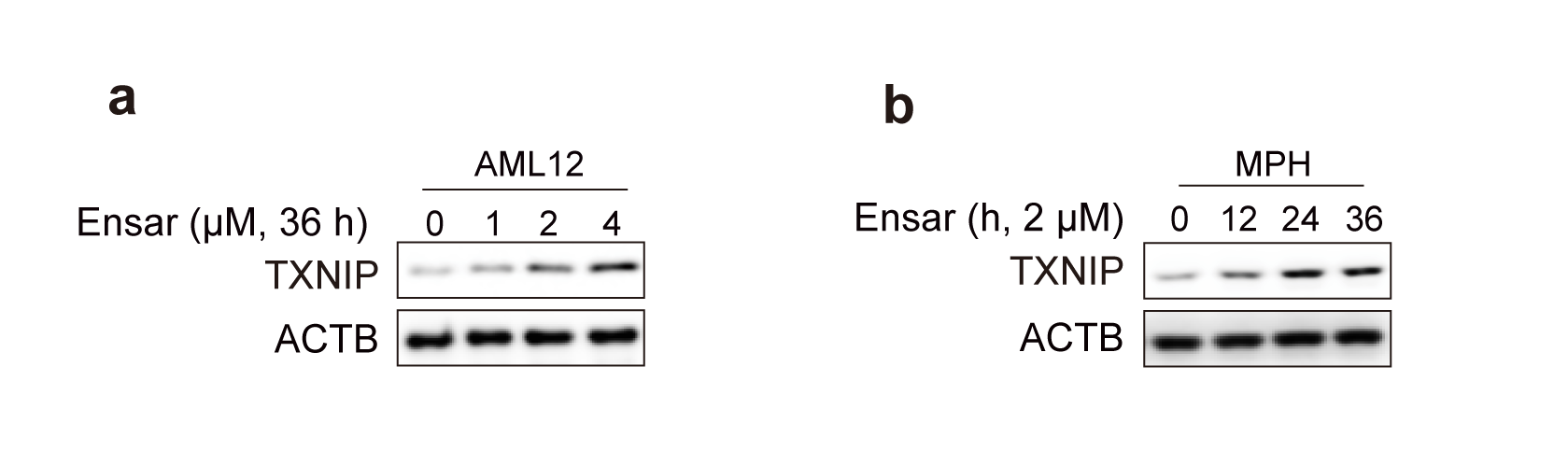


**Fig. S4 Ensartinib up-regulates TXNIP expression in AML12 and mouse primary hepatocytes. (a)** AML12 cells were treated with 0, 1, 2 or 4 μM ensartinib for 36 h. The expression level of TXNIP was measured by western blot. **(b)** MPH cells were treated with 2 μM ensartinib for 0, 12, 24 or 36 h. The expression level of TXNIP was measured by western blot. Ensar, ensartinib. MPH, mouse primary hepatocytes.


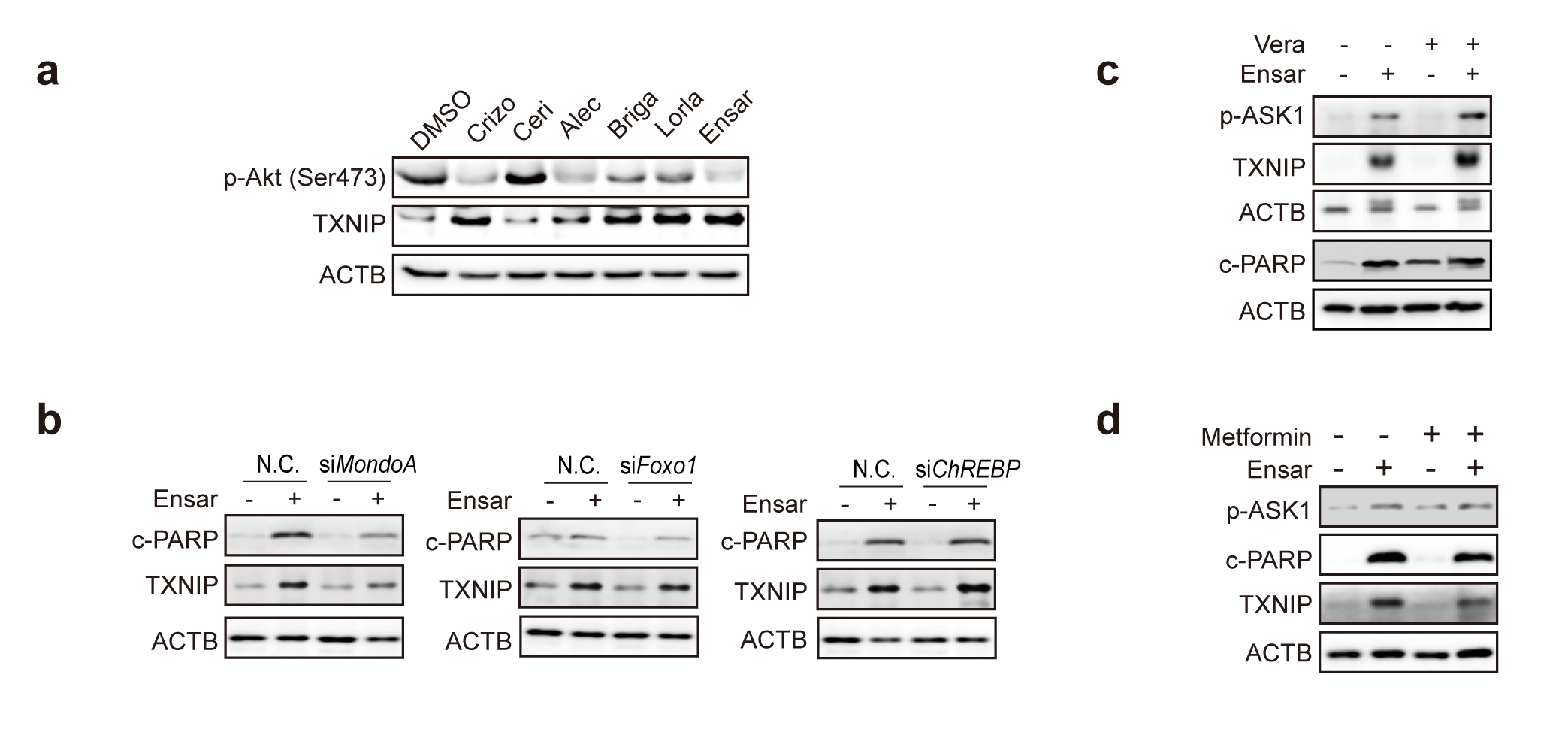


**Fig. S5 Ensartinib upregulates TXNIP and its downstream via the p-Akt (Ser473) and MondoA. (a)** HL-7702 cells were treated with DMSO, 2 fold of Cmax crizotinib, alectinib, brigatinib, lorlatinib and ensartinib for 36 h. The expression levels of p-Akt (Ser473) and TXNIP were measured by western blot. **(b)** NC transfected or *MondoA*, *Foxo1*, *ChREBP*-knockdown HL-7702 cells were treated with ensartinib for 36 h. The expression levels of c-PARP and TXNIP were measured by western blot. **(c)** The expression levels of p-ASK1, c-PARP and TXNIP of HL-7702 cells treated with 2 μM ensartinib and/or 0.5 mM metformin were measured by western blot. **(d)** The expression levels of p-ASK1, c-PARP and TXNIP of HL-7702 cells treated with 2 μM ensartinib and/or 50 μM verapamil were measured by western blot. Ensar, ensartinib. Crizo, crizotinib. Ceri, ceritinib. Alec, alectinib. Briga, brigatinib. Lorla, lorlatinib. Vera, verapamil.


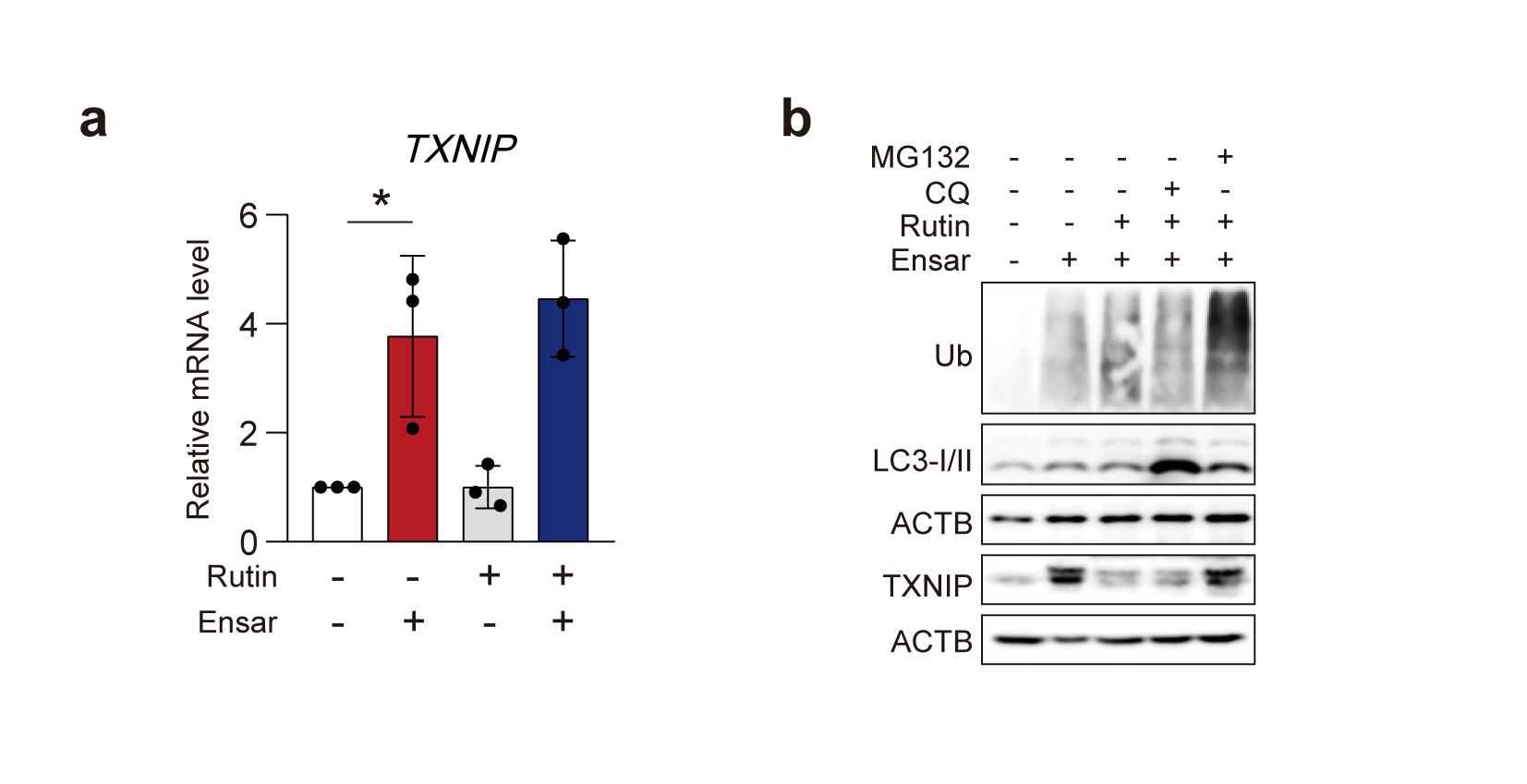


**Fig. S6 Rutin down-regulates TXNIP levels in a transcription-independent manner. (a)** HL-7702 cells were treated with 2 μM ensartinib or/and 5 μM rutin for 36 h. The mRNA expression levels of TXNIP were measured by qPCR. n = 3 independent experiments. **(b)** HL-7702 cells were treated with 2 μM ensartinib or/and 5 μM rutin, 10 μM CQ, 10 μM MG132. The expression levels of Ub, LC3-I/II and TXNIP were measured by western blot. Data were expressed as mean ± SD. **P* < 0.05. One way ANOVA followed by Tukey post hoc test for **(a)**. Ensar, ensartinib. CQ, chloroquine.


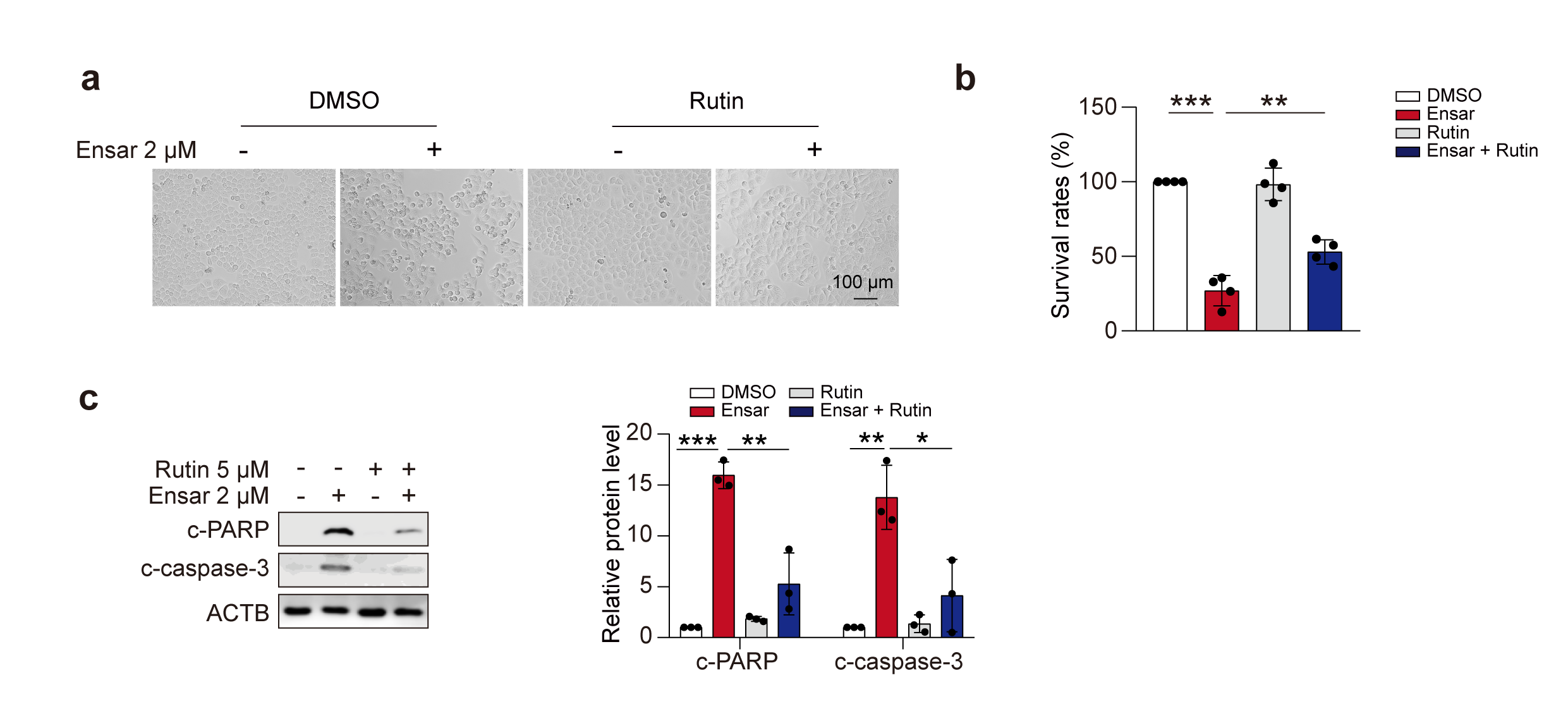


**Fig. S7 Rutin alleviates ensartinib-induced hepatocyte apoptosis.** Ensartinib up-regulates the transcription level of TXNIP. **(a-c)** HL-7702 cells were treated with 2 μM ensartinib or/and 5 μM rutin for 36 h. **(a)** The morphologic changes (original magnification: 200×) were observed by optical light microscope. Scale bar: 100 µm. **(b)** The survival rate of HL-7702 cells treated were measured by SRB staining. n = 4 independent experiments. **(c)** The expression levels of c-PARP and c-caspase-3 were measured by western blot. n = 3 independent experiments. Data were expressed as mean ± SD. **P* < 0.05, ***P* < 0.01, ****P* < 0.001. One way ANOVA followed by Tukey post hoc test for **(b)** and **(c)**. Ensar, ensartinib.


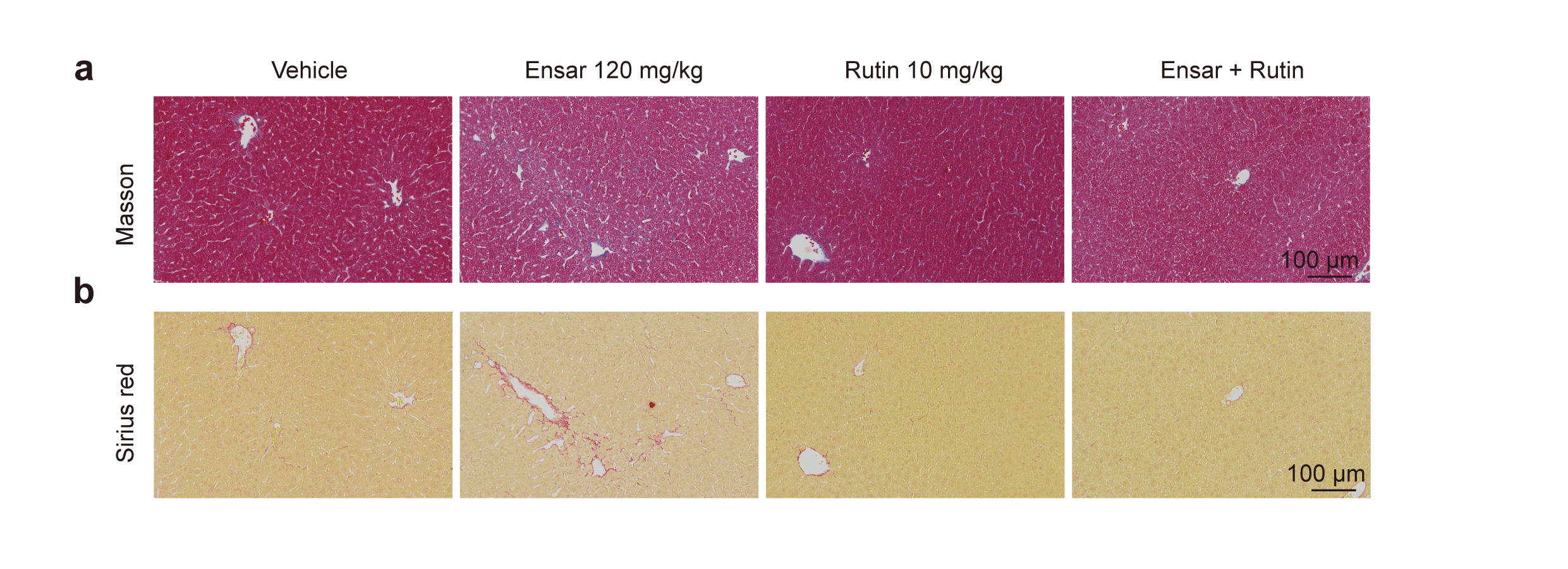


**Fig. S8 Rutin alleviates Ensartinib-induced liver fibrosis *in vivo*.** **(a-b)** C57BL/6J male mice were treated with 120 mg/kg/day ensartinib and/or 10 mg/kg/day rutin for 4 weeks. Representative images (original magnification: 200×) of Masson’s staining **(a)** and Sirius Red staining **(b)** in liver tissues from C57BL/6J mice. Scale: 100 µm. Ensar, ensartinib.


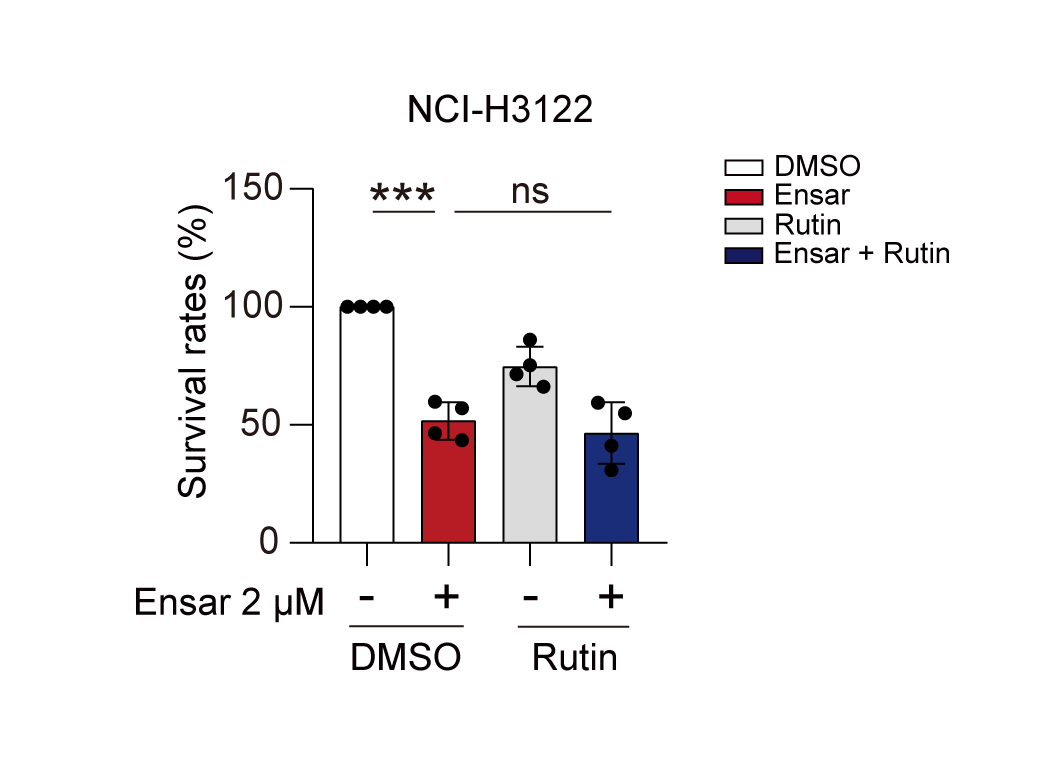


**Fig. S9 Rutin did not attenuate the anti-tumor effect of ensartinib.** NCI-H3122 cells were treated with 2 µM ensartinib and/or 5 µM rutin for 36 h. The survival rates were measured by SRB staining. n = 4 independent experiments. ****P* < 0.001. ns = no significance. Data were expressed as mean ± SD. **P* < 0.05. One way ANOVA followed by Tukey post hoc test. Ensar, ensartinib.

**Table S1. Comparison table of figure 6 compound screening**

| No. | Name | CAS |
| --- | --- | --- |
| 1 | baicalin | 21967-41-9 |
| 2 | hesperetin | 520-33-2 |
| 3 | icariin | 489-32-7 |
| 4 | luteolin | 491-70-3 |
| 5 | tilianin | 4291-60-5 |
| 6 | polydatin | 27208-80-6 |
| 7 | procyanidin B2 | 29106-49-8 |
| 8 | resveratrol | 501-36-0 |
| 9 | rutin | 153-18-4 |
| 10 | salidroside | 10338-51-9 |
| 11 | silibinin | 22888-70-6 |
| 12 | neferine | 2292-16-2 |

Table S2. Antibodies used for western blotting, immunohistochemistry and immunofluorescence

| **Antibody** | **Company** | **Catalogue No.** | **Reactivity** | **Application used in this study** | **Observed Molecular weight (KDa)** |
| --- | --- | --- | --- | --- | --- |
| beta Actin | Diagbio | db7283 | H, M | Western blot | 42 |
| TXNIP | Huabio | ET1705-72 | H, M | Western blot; IHC; IF | 44 |
| Cleaved PARP | Huabio | ET1608-10 | H | Western blot | 89 |
| Cleaved PARP | Abcam | ab32064 | M | Western blot | 25, 89 |
| cleaved caspase-3 (Asp175) | Cell Signaling Technology | #9661 | H, M | Western blot; IHC | 17, 19 |
| phospho-ASK1 | ABclonal | AP1215 | H | Western blot | 155 |
| γ-H2AX | Cell Signaling Technology | #9718 | H | Western blot; IF | 15 |
| Ub (FL-76) | Santa Cruz Biotechnology | sc-9133 | H | Western blot | 10 |
| p-Akt (S473) | ABclonal | AP1208 | H | Western blot | 60 |
| LC3 A/B | Medical & Biological Laboratories | M186-3 | H | Western blot | 15 |
| Tomm20 | Santa Cruz Biotechnology | sc-17764 | H, M | IF | 20 |
